# Mechanisms of Chain Scission under High Strain Rate Sonication in PS–PIB–PS Triblock Copolymers

**DOI:** 10.1021/acsapm.6c02287

**Published:** 2026-08-17

**Authors:** Parth K. Vagholkar, Alexander J. Rosario, John F. Searles, Lisa K. Kemp, Yoan C. Simon, Robson F. Storey, Boran Ma, Dane N. Wedgeworth, Travis Thornell, Sarah E. Morgan

**Affiliations:** † School of Polymer Science and Engineering, 5104 University of Southern Mississippi, Hattiesburg, Mississippi 39406, United States; ‡ School of Molecular Sciences, 43363 Arizona State University, Tempe, Arizona 85287, United States; § Geotechnical and Structures Laboratory, Engineer Research and Development Center (ERDC), 199011 US Army Corps of Engineers (USACE), Vicksburg, Mississippi 39180, United States

**Keywords:** mechanophore, chain scission, ultrasonication, conformation, block copolymers, fluorescence, strain rate

## Abstract

Impedance mismatch block copolymers, combining rubbery polyisobutylene (PIB) with glassy polystyrene (PS) segments, show promise for the design of high-strain-rate material applications. However, the molecular-level processes of energy absorption and dissipation under high strain rates in the solution state are poorly understood. Using a grafting-from approach, we synthesized a library of well-defined PS–PIB–PS triblock copolymers with and without mechanophores at the block interfaces to evaluate the effects of molecular weight, block composition, and dispersity on chain scission induced by high-strain-rate elongational and shear flows under solution-state sonication. Synthesis of block copolymers without the lowest reaction enthalpy bonds identified by DFT analysis allowed interrogation of the site of chain cleavage. GPC and fluorescence spectroscopy analysis revealed that chain scission did not occur preferentially at the block interface, at the lowest enthalpy, or at the chain center in the triblock copolymers but rather was dictated by chain conformation, plasticity, and physical properties of the sonication medium and that stress concentrated at the interface can be tuned by the length and composition of the blocks. Our findings establish an experimental baseline for understanding the effects of factors such as chain architecture, conformation, and sites of stress concentration on the solution-state response of block copolymers under shear and elongational forces.

## Graphical abstract

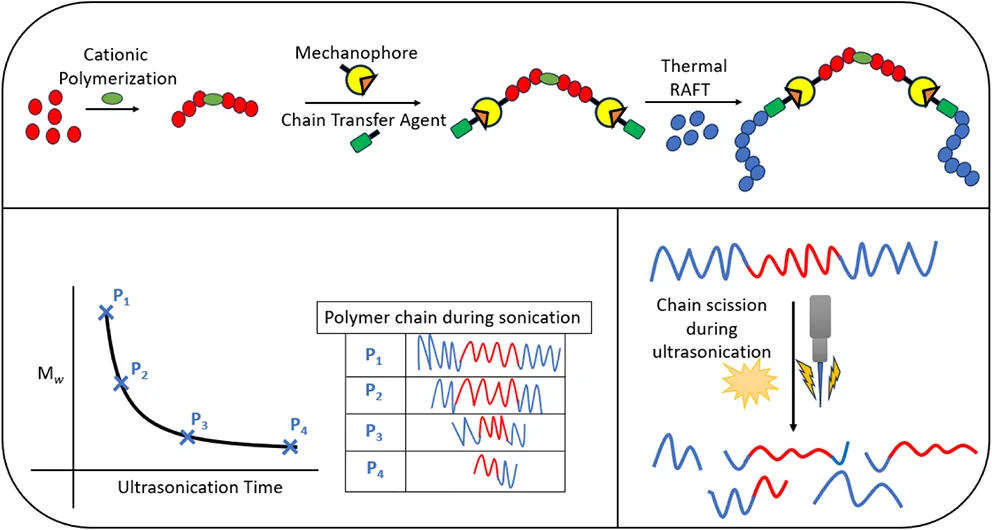

## 1 Introduction

Block copolymers are a versatile class of materials whose properties stem from the covalent linkage of chemically different segments, which can be incorporated in varying sequences and at different block lengths. Polyisobutylene–polystyrene (PIB–PS) diblock copolymers and PS–PIB–PS triblock copolymer thermoplastic elastomers are used in applications including biomedical devices, elastomers, adhesives, and membranes.^1,2^ These impedance-mismatch materials, combining rubbery PIB with glassy PS segments, have potential utility for high-strain-rate applications. A recent report by Luckett et al. showed enhanced performance of composites containing PIB–PS block copolymers under ballistic testing, and a study by Centellas et al. in collaboration with our group reported energy dissipation by both acoustic wave attenuation and plastic deformation in PIB–PS diblock thin films under laser-induced projectile impact test (LIPIT).^3,4^ At this stage, we are only beginning to understand the molecular-level processes of energy absorption and dissipation under high strain rates and their relationship to block copolymer structure.

Chain pullout, chain disentanglement, and chain scission are the principal stress-absorbing mechanisms under high strain rate (10^–2^–10^–7^ s^–1^).^5–7^ The elongational and shear forces created during such high-strain rate events can potentially concentrate the stresses at certain positions on the polymer chain, which can lead to chain scission via rupture of covalent linkages.^8,9^ Characterization of chain scission behavior in the solution state is preferred, as it allows evaluation of molecular properties such as bond energy, chain architecture, molecular weight, and block content, in the absence of complications associated with solid-state morphology, annealing conditions, and processing methods.^10–12^ Solution-state ultrasonication is the most commonly used technique to study the mechanochemical behavior of polymers.^12,13^ The elongational and shear forces created due to cavitation, growth, and eventual implosion of acoustic bubbles are sufficient to stretch the chain and cause localized forces leading to chain scission.^14^ The advantage of ultrasonication lies in its ability to create reproducible chain-scale deformations under high strain rates via acoustic cavitation and, in combination with techniques such as fluorescence spectroscopy and gel permeation chromatography (GPC), determine the areas of stress concentration that result in chain scission.^15,16^

Mechanophores, mechanically labile chemical units that break under shear stress, tension, or compression^17,18^ provide insight into the stress distribution in polymer systems and, in some cases can also serve to redirect and dissipate the force applied to the chain.^19,20^ Classic examples include spiropyrans, which undergo force-induced ring-opening with a chromogenic response, and *gem*-dichlorocyclopropanes (*g*DCC), which activate via conrotatory ring opening.^21,22^ More recently, anthracene–maleimide cycloadducts have gained attention as mechanophores with bright fluorescence yields upon force-activated retro-Diels–Alder cleavage.^23–25^ In homopolymer chains incorporating mechanophores, a promising amount of mechanophore activation has been observed under solution-state ultrasonication, which has allowed stress visualization throughout the chains, mapping centered vs off-center mechanophore activation in polymers.^26–28^

Mechanophores reveal different types of scission behavior depending on the composition and architecture of the polymer system. For example, some studies suggest that chain scission in poly­(methyl acrylate) (PMA) homopolymers occurs at the chain center, while other researchers have reported off-center cleavage of PMA chains incorporating *g*DCC mechanophores.^29,30^ In many cases, the mechanophore is the lowest reaction enthalpy link in the chain, and stress may be concentrated at that point.^31^ Our group showed low mechanophore activation (1.2%) in ultrasonication when anthracene–maleimide mechanophores were placed at PIB–PS graft copolymer junctions, indicating that the primary mode of scission did not occur at the mechanophore linkage in this system.^32^ Wang et al. showed higher mechanophore activation in aggregated PMMA-poly­(butyl acrylate) (PBA) graft copolymers using anthracene–maleimide mechanophores in the self-assembled state.^33^

High strain rate behavior of linear triblock copolymers in the solution state has been the subject of recent computational studies.^34–36^ Zhang et al. suggested that chain scission occurs at the interface of mechanophore-containing PMMA–PBA block copolymers.^35^ Another computational study by Chongvimansin et al. indicated that plasticity and shear factors play a more important role in chain scission during ultrasonication than the strength of the bonds.^36^ To our knowledge, however, there are no experimental reports of chain scission of linear triblock copolymers under sonication.

We present a systematic study of mechanophore-containing triblock copolymers to determine the effects of molecular weight, block content, low enthalpy bonds, and physical factors on force transduction throughout the chain in the solution state. A library of PS–PIB–PS triblock copolymers was synthesized both with and without anthracene–maleimide mechanophores at the block interfaces. Grafting-from methods were employed to synthesize triblock copolymers with varying molecular weights and PIB:PS ratios. Density functional theory (DFT) analysis was conducted to determine the standard reaction enthalpies of each bond on the polymer chain. Solution ultrasonication studies were used to evaluate the effects of molecular weight and block length on mechanophore activation and the rate of chain scission. Gel permeation chromatography (GPC) and fluorescence spectroscopy were used to analyze molecular weight degradation and mechanophore activation, and the rate of molecular weight degradation was calculated using the Baramboim kinetic equation. Chain scission was further analyzed under different solvents.

## 2 Experimental Section

### 2.1 Materials

All materials were used as received, unless otherwise designated.

Isobutylene (IB, BOC Gases) and methyl chloride (Gas and Supply) were dried by passing the gaseous reagent through a column of CaSO_4_/molecular sieves/CaCl_2_ and condensing within a N_2_-atmosphere glovebox immediately prior to use. Titanium tetrachloride (TiCl_4_), dodecanethiol, pentane, 2,6-lutidine, 2-chloro-2,4,4-trimethylpentane (TMPCl), potassium carbonate (K_2_CO_3_), 18-crown-6, sodium chloride, 2,2′-azobis­(2-methylpropionitrile) (AIBN), polystyrene standard (*M*_n_ = 105 kg/mol, *M*_w_ = 110 kg/mol, *Đ =* 1.05), 2,6- di-*tert*-butyl-4-methylphenol (BHT), and maleimide were purchased from Sigma-Aldrich. Acetone, (3-bromopropoxy)­benzene, carbon disulfide (CS_2_), ethyl acetate (EtOAc), anhydrous magnesium sulfate (MgSO_4_), dichloromethane (DCM), sodium bicarbonate (NaHCO_3_), tetrahydrofuran (THF), hydrochloric acid (HCl), methanol, *n*-heptane, dimethylformamide (DMF), toluene, methylcyclohexane, chloroform-*d* (CDCl_3_), and *n*-hexane (95+% extra pure) were purchased from Fisher Scientific. Potassium phosphate (K_3_PO_4_) and 9-anthracenemethanol were purchased from Ark Pharm. *N*-(3-(dimethylamino)­propyl)-*N*′-ethylcarbodiimide hydrochloride (EDC·HCl), *N,N′*-dicyclohexylcarbodiimide (DCC), 2-bromo-2-methylpropionic acid, and 4-dimethylaminopyridine (DMAP) were purchased from Oakwood Chemical.

The difunctional cationic initiator, 5-*tert*-butyl-1,3-di­(1-chloro-1-methylethyl)­benzene (bDCC), was prepared using a previously published procedure.^37^

### 2.2 Instrumentation

#### 2.2.1 Gel-Permeation Chromatography (GPC)

GPC measurements were performed on an ECOSEC HLC-8320GPC TOSOH system consisting of a refractive index detector fitted with two PLgel mixed D columns (pore size range 50–103 Å, 3 μm bead size). Freshly distilled THF served as the mobile phase and was delivered at a flow rate of 0.5 mL/min. The molecular weights were determined from the refractive index detector response using narrow molecular weight PS calibration standards. The lower molecular weight detection limit of our column was found to be below 70 g/mol; it was possible to consistently detect ethyl vinyl ether (72 g/mol) during polymer synthesis, indicating that low molecular weight fragments/oligomers produced during sonication can be effectively detected.

#### 2.2.2 Attenuated Total Reflection Fourier Transform Infrared Spectroscopy (ATR-FTIR)

Living carbocationic polymerizations (LCP) of isobutylene and styrene were monitored via real-time ATR-FTIR using a Mettler Toledo ReactIR 4000, equipped with a diamond composite probe and integrated with an N_2_-atmosphere glovebox. Reduction in the area of the peaks at 887 cm^–1^ (=CH_2_ wag of isobutylene) and 907 cm^–1^ (wag of the vinyl group in styrene) indicated the formation of the PIB midblock and PS end blocks, respectively.

#### 2.2.3 Nuclear Magnetic Resonance (NMR) Spectroscopy

NMR spectra were obtained at 25 °C in CDCl_3_ with a 400 MHz Bruker Avance III (TopSpin 3.1p17) spectrometer by taking an average of 32 scans (delay 2 s). Chemical shifts for ^1^H NMR spectra were referenced to signals from CDCl_3_ (δ = 7.26 ppm).

#### 2.2.4 Pulsed Ultrasound

A 2 mg/mL solution of the triblock copolymer with added BHT (30 mM) as a radical inhibitor was prepared in 40 mL of freshly distilled THF for ultrasonication. The polymer solution was added to a sealed two-arm jacketed Suslick flask and cooled to 5 °C using a chiller. The sonication horn, with a microtip diameter of 0.5 in, was placed in the solution at a distance of 2 cm from the bottom of the cell. The solution was then sparged with nitrogen for 15 min after which the needle was removed, and a nitrogen-filled balloon was attached via a syringe through the rubber septum to provide positive pressure throughout the sonication experiments. Sonication was performed at 20% amplitude using a pulse sequence of 1 s on/1 s off. Under these conditions and geometry, the delivered power is 13.91 W and the generated strain rate is estimated to be 10^5^–10^6^ s^–1^, high enough to cleave the chains due to imploding bubbles. THF was chosen as the solvent because of its low viscosity in comparison to water (reducing viscous damping). The solution was chilled to 5 °C both to minimize local hot spots and to reduce solvent vapor pressure, allowing rapid implosion of bubbles and maximizing the local strain rate. The relatively large horn diameter in the small volume of solution results in an even distribution of strain rate, in the range of 10^5^–10^6^ s^–1^.^7,38,39^ Aliquots (3 mL) were taken at regular intervals by syringe for GPC and fluorometer analysis.

#### 2.2.5 Fluorescence Spectroscopy

The mechanophore activations of block copolymers (during ultrasonication) were determined by the concentration of free anthracene groups using fluorescence spectroscopy. Emission scans were performed using an excitation wavelength (λ_exc_) of 356 nm and an emission wavelength (λ_em_) of 380–500 nm on a HORIBA QM 400 fluorometer with a 1 cm standard quartz cell. The mechanophore activation was determined by fitting the intensity value to the standard fluorescence calibration curve as explained by Mehringer et al.^32^ Briefly, a calibration curve was obtained for an anthracene-functionalized polystyrene (PS-Anth) (λexc = 356 nm, λem = 414 nm), with intensity plotted as a function of concentration (Figure S1). Mechanophore activation of the triblock copolymers was determined by evaluating the fluorescence spectroscopy of aliquots taken during and after ultrasonication. Percent mechanophore activation was determined by comparing the intensity of the released anthracene moiety during sonication to the intensity at 100% activation from the calibration curve (Table S1). Each sample was evaluated in triplicate and the average % activation ± SD is reported.

Low temperature (5 °C), 20% amplitude, a radical inhibitor, and an N_2_ atmosphere were employed for sonication to avoid excessive transient heat which could lead to side reactions, such as Diels–Alder coupling of the cleaved mechanophore. To evaluate the possibility of this reaction under our sonication conditions, a solution of PIB-MI (54 kg/mol) with PS-Anth (65 kg/mol) was ultrasonicated for 3 days, and aliquots were analyzed by fluorescence spectroscopy to determine if anthracene intensity was reduced due to consumption in the Diels–Alder reaction (Figure S2). The fluorescence intensity remained constant throughout the 3-day experiment, indicating that no reaction between the cleaved mechanophores took place. Similar behavior was observed in our previous report, where the reaction of anthracene–maleimide adducts required extended reaction time (>5 days) at high temperature (120 °C) for appreciable yield.^32^ We assume that the steric hindrance of the large molecular weight chains, insufficient mechanochemical stress at the chain ends (where the mechanophores are present after cleavage), and insufficient thermal energy during sonication for the concerted Diels–Alder reaction lead to the absence of recombination of the cleaved MI-PIB and PS-Anth moieties during sonication.

### 2.3 General Procedure for Synthesis of Mechanophore-Free PS–PIB–PS Triblock Copolymer

Following a previously published procedure with modifications,^40^ the reaction was conducted in a 500 mL 4-necked round-bottom flask (RBF) equipped with a mechanical stirrer, a ReactIR probe, and two addition funnels in the necks. The flask was submerged in a heptane bath inside an N_2_-atmosphere glovebox under moisture-free and oxygen-free conditions. Liquid nitrogen was used to maintain the temperature at −75 °C in the heptane bath. To the reaction vessel were added methyl cyclohexane (60 mL, 0.47 mol), dichloromethane (40 mL, 0.63 mol), bDCC (87.94 mg, 0.31 mmol), 2,6-lutidine (0.054 mL, 0.46 mmol), and isobutylene (16.91 mL, 0.21 mol). The reaction was then started by the addition of TiCl_4_ (0.67 mL, 6 mmol), and the conversion of IB was tracked by the disappearance of the 887 cm^–1^ peak in the ReactIR. Upon reaching 99% IB conversion, a mixture of methyl cyclohexane (11 mL, 0.08 mol), DCM (7 mL, 0.1 mol), and styrene (13 mL, 0.12 mol) was added to the flask to initiate polystyrene synthesis. The styrene conversion was monitored by the peak at 907 cm^–1^, and the reaction was continued until 35% conversion of styrene, when cold methanol (0 °C) was added (80 mL) under stirring to quench it. The RBF was then taken out of the glovebox, and the solvents were evaporated under vacuum. The crude product was dissolved in THF and precipitated thrice in room temperature (RT) methanol under stirring. The product was vacuum filtered and subsequently dried in a vacuum oven at 40 °C for 2 days. The molecular weight of the triblock was analyzed via GPC, and the weight fraction of blocks in the copolymer was determined via NMR. A library of triblock copolymers with varying molecular weights and PS weight fractions was synthesized in 75% yield following this procedure.

### 2.4 Synthesis of Mechanophore-Containing PS–PIB–PS Triblock Copolymer

#### 2.4.1 Difunctional PIB Bromine (Br-PIB-Br)

Following a previously published procedure with modifications (in temperature and catalyst loading),^40^ a 500 mL 4-necked RBF equipped with a mechanical stirrer, ReactIR probe, and addition funnels was submerged in a heptane bath in a N_2_-atmosphere glovebox under air-free and oxygen-free conditions. Liquid nitrogen was used to maintain the temperature at −55 °C in the heptane bath. To the reaction vessel were added *n*-hexane (44 mL, 0.34 mol), methyl chloride (65 mL, 1.28 mol), bDCC (143.64 mg, 0.51 mmol), 2,6-lutidine (0.05 mL, 0.43 mmol), and isobutylene (23 mL, 0.28 mol). The reaction was then started by the addition of TiCl_4_ (0.25 mL, 2.29 mmol), and the conversion of IB was tracked via the peak at 887 cm^–1^ in the ReactIR. Upon reaching 99% isobutylene conversion, (3-bromopropoxy)­benzene (2.35 mL, 14.9 mmol) was added, and the reaction was brought to −72 °C. TiCl_4_ (3.27 mL, 29.8 mmol) was then injected into the flask, and the reaction was stirred overnight until termination by the addition of excess cold (0 °C) methanol. The resulting solution was warmed to room temperature and then precipitated into methanol. The precipitate was collected, dissolved in *n*-hexane, and reprecipitated in methanol. The precipitate was dissolved in *n*-hexane and washed twice with deionized water followed by a saturated sodium chloride solution, dried over MgSO_4_, filtered, and vacuum-stripped in a rotary evaporator followed by drying in *vacuo* to yield the isolated polymer (80% yield). Two bromine-functional PIB homopolymers with weight-average molecular weight (*M*_w_) = 30 kg/mol, dispersity (*Đ)* = 1.13 (Br-PIB_30_-Br) and *M*_w_ = 45 kg/mol, *Đ* = 1.12 (Br-PIB_45_-Br) were synthesized using this procedure in order to make macroinitiators for thermally reversible addition–fragmentation chain transfer (RAFT) polymerization by the following synthesis steps.

#### 2.4.2 Difunctional PIB Oxanorbornene Imide (Fur-MI-PIB_45_-MI-Fur)

Br-PIB_45_-Br (15 g, 0.35 mmol) was dissolved in THF (200 mL) and DMF (40 mL); *exo*-7-oxanorbornene-2,3-dicarboximide (1.14 g, 6.95 mmol), K_2_CO_3_ (1.19 g, 8.57 mmol), and 18-crown-6 (0.93 g, 3.5 mmol) were added to the stirring solution. The mixture was heated at 50 °C for 12 h under a nitrogen atmosphere. Upon completion, the THF was removed by rotary evaporation, and the remaining product was dissolved in hexanes, filtered, and precipitated by slowly adding it into a large excess of methanol. The polymer was collected, dissolved in hexanes, washed 3× with distilled water, dried over MgSO_4_, and vacuum-stripped using a rotary evaporator, followed by drying *in vacuo* to obtain the final product (11.2 g, 75% yield, *M*_w_ = 45.8 kg/mol, *Đ* = 1.14).

#### 2.4.3 Difunctional PIB Maleimide (MI-PIB_45_-MI)

Fur-MI-PIB_45_-MI-Fur (10 g, 0.22 mmol) was dissolved in toluene (100 mL), and BHT was added as a radical inhibitor (30 mmol) in a 250 mL RBF equipped with a magnetic stirrer. The mixture was heated at 110 °C for 12 h under a nitrogen atmosphere. Upon completion, the toluene was removed by rotary evaporation, and the remaining product was dissolved in hexanes, precipitated by slowly adding it into a large excess of methanol, and then filtered. The maleimide-functional PIB was collected, dissolved in hexanes, washed three times with distilled water, dried over MgSO_4_, and vacuum-stripped at room temperature to obtain the final product (70% yield, *M*_w_ = 46.1 kg/mol, *Đ* = 1.14).

#### 2.4.4 Synthesis of 2-(((Dodecylthio)­carbonothioyl)­thio)-2-methylpropanoic Acid (DMP)

Following a previously published procedure,^41^ acetone (180 mL) and K_3_PO_4_ (12.78 g, 59.3 mmol) were added to a 500 mL flame-dried RBF and put under an N_2_ atmosphere. Dodecanethiol (14.2 mL, 59.3 mmol) was added to the solution over 15 min using an addition funnel, and the mixture was allowed to stir for an additional 5 min. CS_2_ (9.67 mL, 160.1 mmol) was then added dropwise, causing the solution to turn a yellow-orange color. After stirring for 1 h, 2-bromo-2-methylpropionic acid (8.82 g, 52.8 mmol) was added to the flask, and the reaction was allowed to stir overnight. The next day, the mixture had solidified into a solid yellow paste. The paste was dissolved with EtOAc and washed with HCl (1 M), followed by a brine wash. The organic phase was dried overnight with MgSO_4_, which was subsequently removed by filtration. The filtrate was concentrated via rotary evaporation and then purified via gradient column chromatography (5 → 10% EtOAc in hexanes) to yield a yellow crystalline solid (16.6 g, 86.2% yield).

#### 2.4.5 Synthesis of Anthracen-9-ylmethyl DMP (AMDMP)

Following a previously published procedure,^41^ DMP (16.55 g, 45.4 mmol), EDC·HCl (13.05 g, 68.1 mmol), DMAP (8.32 g, 68.1 mmol), and DCM (100 mL) were combined in an oven-dried 500 mL 3-neck RBF to yield a heterogeneous orange solution with a white solid. A solution of 9-anthracenemethanol (14.23 g, 68.1 mmol) in DCM (50 mL) was added to the flask, and the mixture was stirred overnight under N_2_. The reddish-brown solution was washed with NaHCO_3_ (1 M), followed by HCl (1 M), and finally with brine. After the washes, the dark red solution was dried with MgSO_4_ and filtered to remove any solids. The filtrate was concentrated via rotary evaporation and purified via column chromatography (6% EtOAc in hexanes) yielding a yellow crystalline solid (14.7 g, 58.6% yield).

#### 2.4.6 Trithiocarbonate Difunctional PIB (AMDMP-PIB-AMDMP)

To a 250 mL RBF (equipped with a magnetic stirrer) were charged 7 g (0.15 mmol) of difunctional maleimide-terminated PIB (MI-PIB-MI) dissolved in toluene (70 mL) and 280 mg (0.75 mmol) of AMDMP, and the resulting solution was sparged with N_2_ for 30 min. The reaction solution was then heated to 100 °C and stirred for 6 h. The solution was subsequently concentrated at room temperature under vacuum, dissolved in THF, precipitated in a cold mixture of 50:50 acetone:methanol, and centrifuged for 5 min at 2800 rpm. The supernatant was discarded, and the PIB which precipitated at the bottom of the centrifuge tube was collected with a spatula, dried *in vacuo* overnight at 50 °C in a scintillation vial, and stored in dark (yield= 60%, *M*_n_ = 47.0 kg/mol, *Đ* = 1.14).

#### 2.4.7 Thermal RAFT Polymerization (PS–PIB–PS)

AMDMP-PIB-AMDMP (225 mg, 5 × 10^–6^ mol) was dissolved in styrene (5 mL, 0.043 mol) and toluene (5 mL), followed by the addition of 0.3 mL of an AIBN stock solution (11 mg AIBN in 11 mL toluene) into a scintillation vial with a pressure relief septum cap and equipped with a magnetic stirrer. The reaction mixture was sparged with N_2_ for 45 min before holding it on a heating block at 85 °C to initiate the polymerization. Aliquots (0.2 mL) were removed by syringe at regular time intervals to monitor the progress of the reaction via GPC. Upon reaching the desired molecular weight, the vial was frozen by placing it in a liquid N_2_ dewar followed by opening the vial to the atmosphere. After returning to room temperature, toluene was removed via a rotary evaporator. The concentrate was then dissolved in THF, precipitated thrice in cold methanol (0 °C), filtered, and dried overnight *in vacuo* at 50 °C to yield a solid product. A library of triblock copolymers containing mechanophores, with varying molecular weights and weight percentages of PS, was synthesized using this procedure.

### 2.5 General Procedure for Synthesis of PIB Homopolymer

Following a previously published procedure with modifications (in solvent type and monomer concentration),^42^ a 500 mL 4-necked-RBF equipped with a mechanical stirrer, ReactIR probe, and addition funnels was submerged in a heptane bath in a glovebox under air-free and oxygen-free conditions. Liquid nitrogen was used to maintain the temperature at −55 °C in the heptane bath. To the reaction vessel were added *n*-hexane (44 mL, 0.34 mol), methyl chloride (65 mL, 1.28 mol), bDCC (143.64 mg, 0.51 mmol), 2,6-lutidine (0.05 mL, 0.43 mmol), and isobutylene (23 mL, 0.28 mol). The reaction was then started by the addition of TiCl_4_ (0.25 mL, 2.29 mmol), and the conversion of IB was tracked via the peak at 887 cm^–1^ in the ReactIR. Upon reaching 99% isobutylene conversion, the reaction was terminated by the addition of 80 mL of methanol. The resulting solution was warmed to room temperature and then precipitated into methanol. The precipitate was collected, dissolved in *n*-hexane, and reprecipitated in methanol. The precipitate was dissolved in *n*-hexane and washed twice with deionized water followed by a saturated sodium chloride solution, dried over MgSO_4_, filtered, vacuum-stripped in a rotary evaporator, and dried *in vacuo* to yield the isolated polymer (80% yield). Two PIB homopolymers with *M*_w_= 110 kg/mol, dispersity (*Đ)* = 1.18 and *M*_w_= 45 kg/mol, *Đ* = 1.12 were synthesized using this procedure.

### 2.6 General Procedure for Synthesis of PIB–PS Diblock Copolymer

Following a previously published procedure,^4^ the reaction was conducted in a 500 mL 4-necked RBF equipped with a mechanical stirrer, ReactIR probe, and two addition funnels in the necks. The flask was submerged in a heptane bath inside a glovebox under moisture-free and oxygen-free conditions. Liquid nitrogen was used to maintain the temperature at −75 °C in the heptane bath. To the reaction vessel were added methyl cyclohexane (60 mL, 0.47 mol), dichloromethane (40 mL, 0.63 mol), TMPCl (90.84 mg, 0.62 mmol), 2,6-lutidine (0.054 mL, 0.46 mmol), and isobutylene (16.91 mL, 0.21 mol). The reaction was then started by the addition of TiCl_4_ (0.67 mL, 6 mmol), and the conversion of IB was tracked by the disappearance of the IR peak at 887 cm^–1^ in the ReactIR. Upon reaching 99% IB conversion, a mixture of methyl cyclohexane (11 mL, 0.08 mol), DCM (7 mL, 0.1 mol), and styrene (13 mL, 0.12 mol) was added to the flask to initiate polystyrene synthesis. The styrene conversion was monitored by the peak at 907 cm^–1^, and the reaction continued until 35% conversion of styrene. The reaction was then quenched by the addition of 80 mL of cold methanol (0 °C) under stirring. The RBF was then taken out of the glovebox, and the solvents were evaporated under vacuum. The crude product was dissolved in THF and precipitated thrice in methanol under stirring. The product was vacuum filtered and subsequently dried in a vacuum oven at 40 °C for 2 days. The molecular weight of the diblock copolymer was analyzed via GPC, and the weight fraction of blocks in the copolymer was determined via NMR.

### 2.7 Computational Details

All density functional theory (DFT) calculations were performed with ORCA v. 5.0.4 software.^43,44^ Avogadro v. 1.2.0 was used for the visualization of structures.^45^ Open Babel v. 3.1.0 was used for conformation searches.^46^ The initial structures for all reactants and products were obtained through an Open Babel systematic rotor (conformer) search. These initial structures were used as input for the DFT calculations. The DFT calculations used the B3LYP functional^47^ and the aug-cc-pVD­(+d)­Z basis set.^48–52^ Calculations on radical-containing structures were done in an open-shell configuration, while all other calculations were in a closed shell configuration. D3 dispersion corrections were used.^53^ ORCA’s DEFGRID3 was used for the numerical integration grid. Geometry optimization, single-point energy calculations, and analytical frequency calculations were done for all structures of interest. The geometry optimizations were performed with tight tolerances of 2.0e-07 Eh for the energy change, 3.0e-05 Eh/bohr for the maximum gradient, 8.0e-06 Eh/bohr for the RMS gradient, 2.0e-04 bohr for the maximum displacement, and 1.0e-04 for the RMS displacement. The analytical frequency calculations provided thermal corrections to the electronic energy at 1.0 atm pressure and 298.15 K, enabling access to the enthalpy of the molecules.

The DFT computations can be divided into operations on 5 discrete chemical moieties of the copolymer structure shown in Figure S27. These moieties are highlighted in the structure of Figure S27 by the breaking bond(s) in the moiety’s reaction pathway during ultrasonication. The enthalpy of reaction from bond cleavage (or retro-Diels–Alder in the case of the mechanophore) in these 5 regions of interest is tabulated in Table S3, and the specific reactant and product structures used for the calculations are provided in Figures S28–S32. Enthalpies of reaction were calculated using eq S1.

## 3 Results and Discussion

### 3.1 Block Copolymers Synthesized

A library of PS–PIB–PS copolymers, with and without mechanophores at the block junction, was synthesized for the evaluation of chain scission at high strain rates under solution-state sonication. Four triblocks without mechanophores were synthesized using the sequential addition of monomers during LCP (Table 1, entries 1–4). The target *M*_w_ of the PIB midblock was 45 kg/mol, and *M*_w_s of 41–49 kg/mol with *Đ* of 1.04–1.17 were obtained. PIB volume fractions of 30% to 70% were targeted by varying the *M*_w_ of the PS end blocks, achieving triblock copolymers of *M*_w_ 58–121 kg/mol with *Đ* of 1.19–1.64. The polymer naming scheme references the targeted *M*_*w*_ of the blocks; for example, PS_7_–PIB_45_–PS_7_ denotes a triblock copolymer with a PIB midblock *M*_w_ target of 45 kg/mol and two PS end blocks with a target *M*_w_ of 7 kg/mol. GPC traces and NMR spectra of the triblock copolymers are shown in Figures S3, S4, and S5.

**Table 1:** Triblock Copolymers with and without Mechanophores Synthesized with Varying Molecular Weights and PS Content^a^,^b^

Sample Name	*M*_n_ PIB midblock (kg/mol)	*M*_w_ PIB midblock (kg/mol)	*Đ* PIB midblock	*M*_n_ Triblock (kg/mol)	*M*_w_ Triblock (kg/mol)	*Đ* Triblock	Target PIB Volume Fraction	PIB Volume Fraction
PS_7_–PIB_45_–PS_7_	41	46	1.12	48	58	1.19	0.70	0.74
PS_17_–PIB_45_–PS_17_	47	49	1.04	57	74	1.29	0.60	0.62
PS_30_–PIB_45_–PS_30_	37	41	1.14	67	107	1.49	0.40	0.37
PS_38_–PIB_45_–PS_38_	41	48	1.17	74	121	1.64	0.30	0.25
PS_13_-M-PIB_30_-M-PS_13_	27	29	1.08	43	56	1.28	0.60	0.57
PS_22_-M-PIB_30_-M-PS_22_	27	29	1.08	53	75	1.41	0.40	0.40
PS_45_-M-PIB_30_-M-PS_45_	27	29	1.08	75	118	1.57	0.30	0.25
PS_17_-M-PIB_45_-M-PS_17_	41	44	1.06	60	79	1.31	0.50	0.48
PS_37_-M-PIB_45_-M-PS_37_	41	44	1.06	76	120	1.58	0.30	0.29

a Sample name contains the targeted *M*_w_ of the blocks as subscripts. Reported *M*_n_, *M*_w_, and *Đ* were determined by GPC.

b Volume fractions were calculated from NMR integration assuming ρ_PS = 1.00 g/mL and PIB = 0.92 g/mL.

In a second series of triblock copolymers, anthracene–maleimide mechanophores were incorporated at the junction of PIB and PS blocks to allow visualization of chain scission at the PIB–PS interface under high strain rate. A “grafting-from” approach was used to incorporate two mechanophores in each triblock chain, by first synthesizing a telechelic PIB midblock and subsequently forming the outer PS blocks using reversible-deactivation radical polymerization. For this series, “M” denotes the presence of a mechanophore, and subscripts denote the target *M*_w_ of the blocks (Table 1, entries 5–9).

To obtain the PIB macroinitiator, we synthesized a Br-PIB-Br midblock of the desired molecular weight using LCP (Scheme 1A and Figure 1A), which we functionalized with a maleimide–furan adduct by nucleophilic substitution to obtain maleimide–furan-functional telechelic PIB (Fur-MI-PIB-MI-Fur) (Scheme 1B). The appearance of alkene peaks at 6.5 ppm and bridgehead protons at 5.2 and 2.86 ppm in the ^1^H NMR spectrum (Figure 1B) indicates the conversion of the end groups to MI-Fur.^42^ The furan group was subsequently thermally cleaved via a retro-Diels–Alder reaction to expose reactive maleimide groups, yielding MI-PIB-MI, as shown in Scheme 1C. The structure is verified by the complete disappearance of the furan alkene groups at 6.5 ppm and the appearance of the maleimide double bond at 6.7 ppm in the ^1^H NMR spectrum (Figure 1C).

**Scheme 1:**
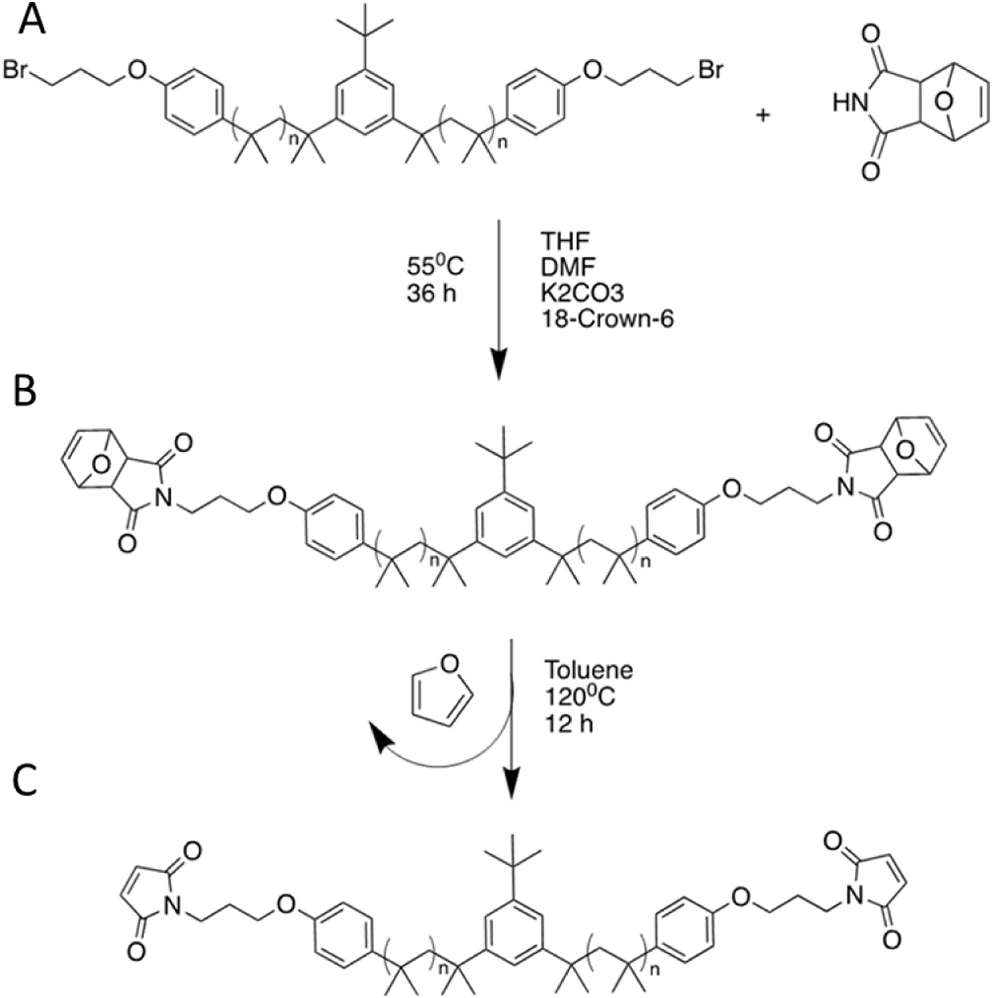
Synthesis of Maleimide Functional PIB: A) Br-PIB-BR, B) Fur-MI-PIB-MI-Fur, C) MI-PIB-MI

**Figure 1:**
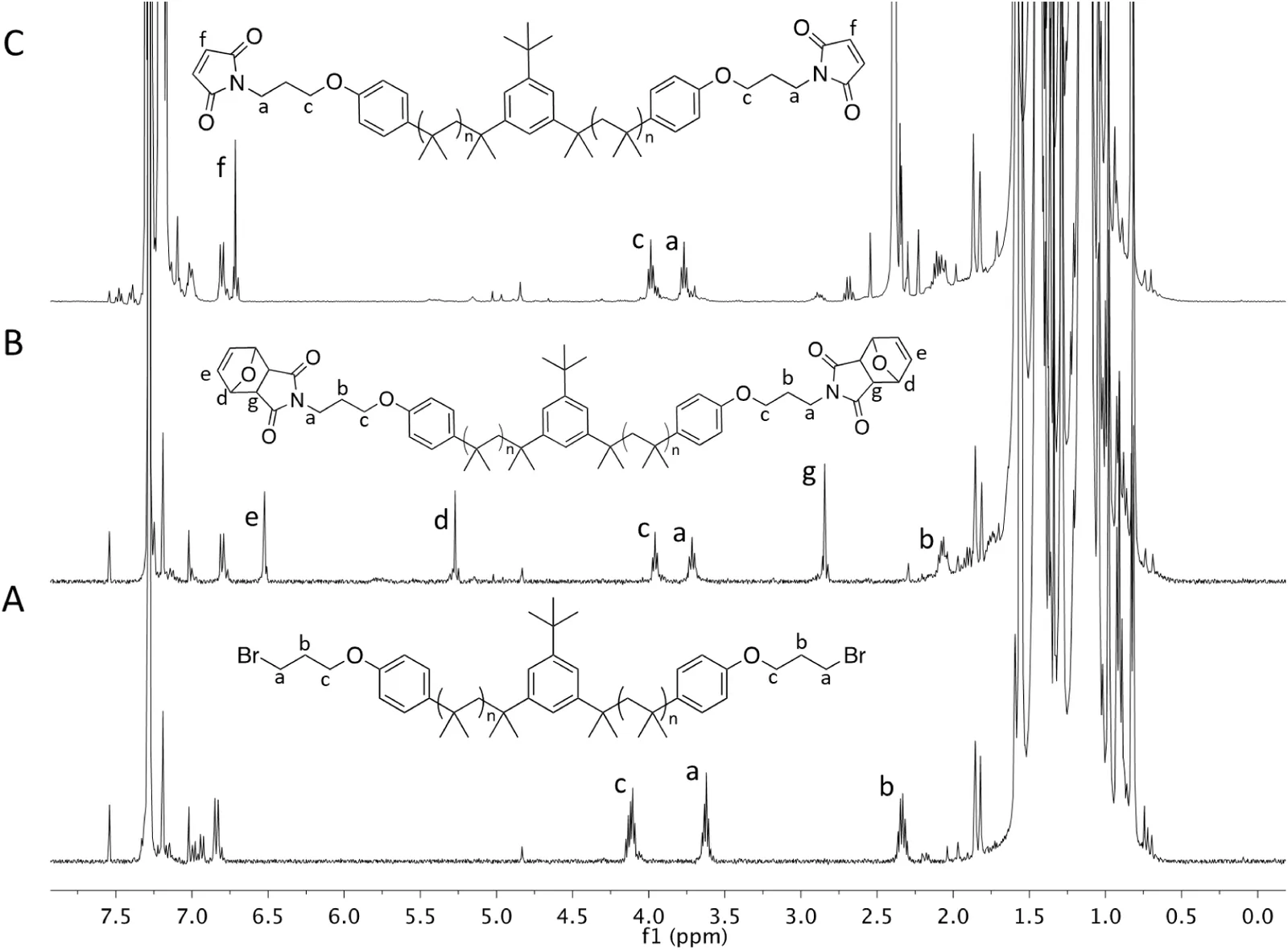
^1^H NMR stack plot showing the formation of maleimide-functional telechelic PIB: A) Br-PIB-Br, B) Fur-MI-PIB-MI-Fur, C) MI-PIB-MI.

An anthracene-functional DMP RAFT agent was synthesized using the procedure described in the experimental section and functionalized via EDC coupling with 9-anthracene methanol (Scheme S1). The presence of the anthracene protons between 7 and 8.5 ppm and methylene protons at 6 ppm (labeled “e”) in Figure S6 demonstrates complete functionalization of the RAFT agent. The small triplet at around 2.69 ppm is due to thiol contaminant still present in the material which does not affect the further reactions in our synthesis.^41,54,55^

The anthracene-functional RAFT agent was then reacted with the maleimide-functional PIB via a Diels–Alder reaction (Scheme 2). The disappearance of the anthracene conjugation (>7.5 ppm), the presence of PIB quencher (labeled “e” at 3.85 ppm, “f” at 3.65 ppm, and “g” at 2.5 ppm), mechanophore bridgehead protons (labeled “a” at 3.4 ppm and “b” at 4.7 ppm), methylene bound to anthracene (labeled “c” between 5.25–5.5 ppm), and methylene adjacent to the thiocarbonate group (labeled “d” at 2.8 ppm) in the ^1^H NMR spectrum in Figure 2 indicate the complete formation of the mechanophore, resulting in the establishment of RAFT moieties at the chain ends (RAFT-PIB-RAFT).

**Scheme 2:**
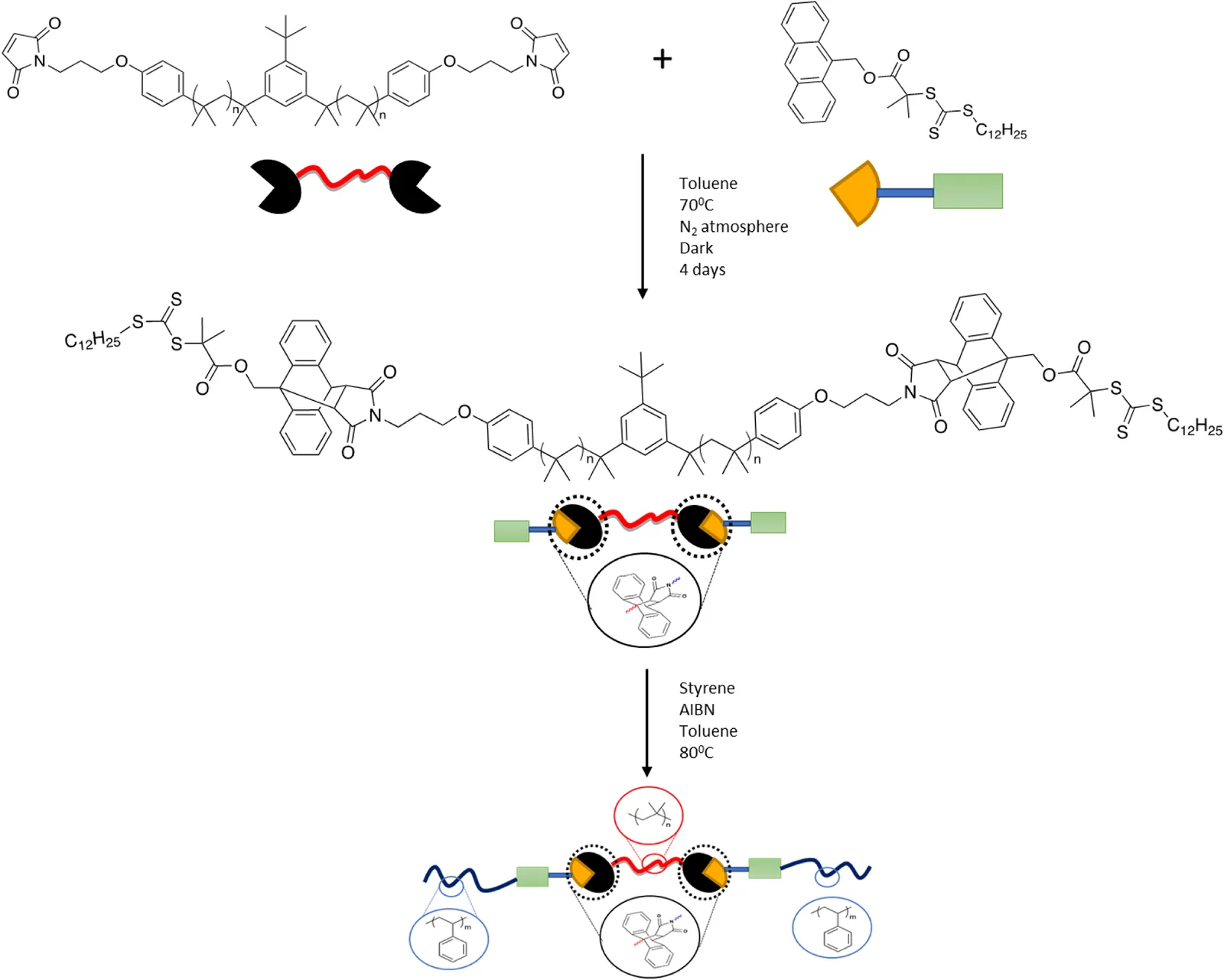
Synthesis of PIB Macroinitiator via Diels–Alder Reaction, and the Mechanophore Containing PS-M-PIB-M-PS via Thermal RAFT Polymerization

**Figure 2:**
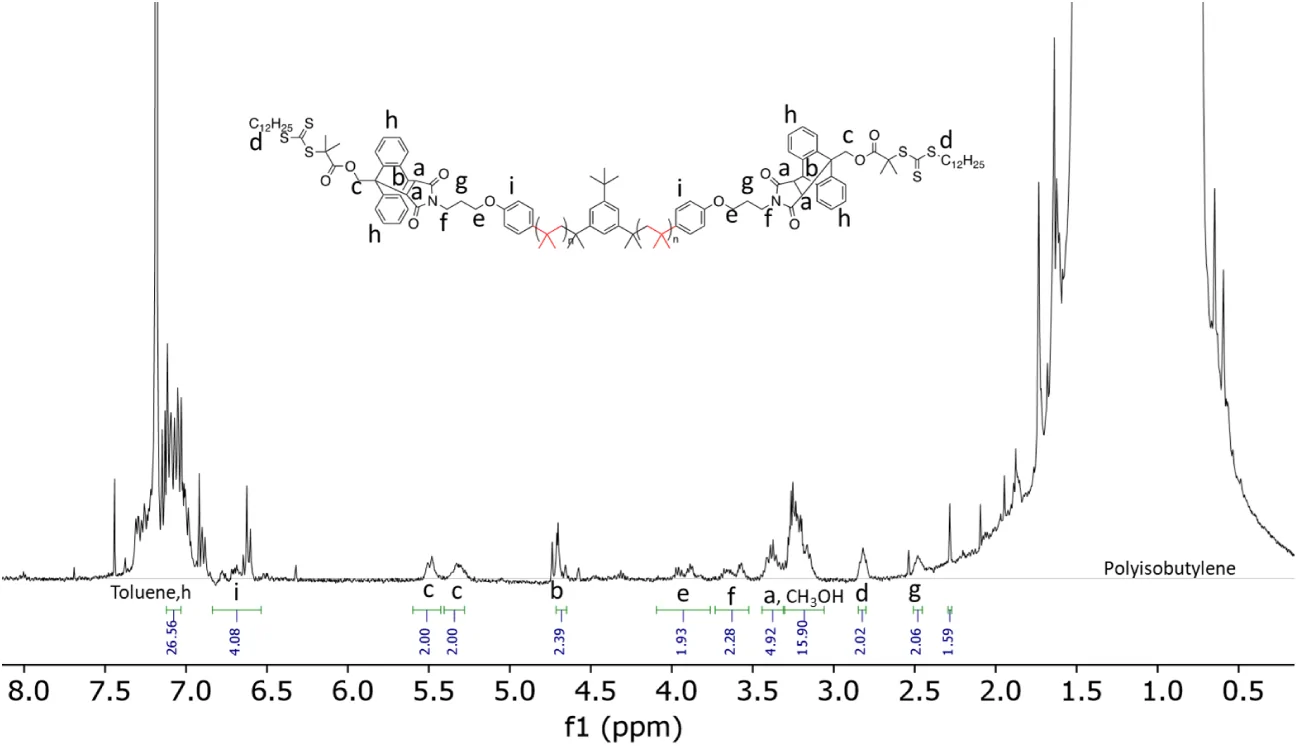
^1^H NMR spectrum showing the successful formation of the PIB macroinitiator with the presence of mechanophore peaks, PIB quencher peaks, and thiocarbonate proton peaks.

PS–PIB–PS triblock copolymers with mechanophores at the junction were synthesized using thermal RAFT polymerization as shown in Scheme 2. Two molecular weights of RAFT–PIB–RAFT macroinitiator center blocks were targeted, 30 and 45 kg/mol, which were subsequently employed to prepare triblocks with molecular weights varying from 56 to 120 kg/mol (Table 1), The mechanophore was not activated during the polymerization, as shown by the presence of bridgehead proton peaks and the absence of any free anthracene peaks in the ^1^H NMR spectrum for PS_13_-M-PIB_30_-M-PS_13_ in Figure 3A. GPC chromatograms displayed monomodal distributions for all polymerizations, as shown for PS_17_-M-PIB_45_-M-PS_17_ in Figure 3B and for others in Figure S7, with increasing molecular weights as the PS block length increased. Two mechanophore-containing triblock copolymers with low *Đ* were prepared by recirculating through a recycling GPC. Comparative molecular weight properties and GPC traces are shown in Figure S8.

**Figure 3:**
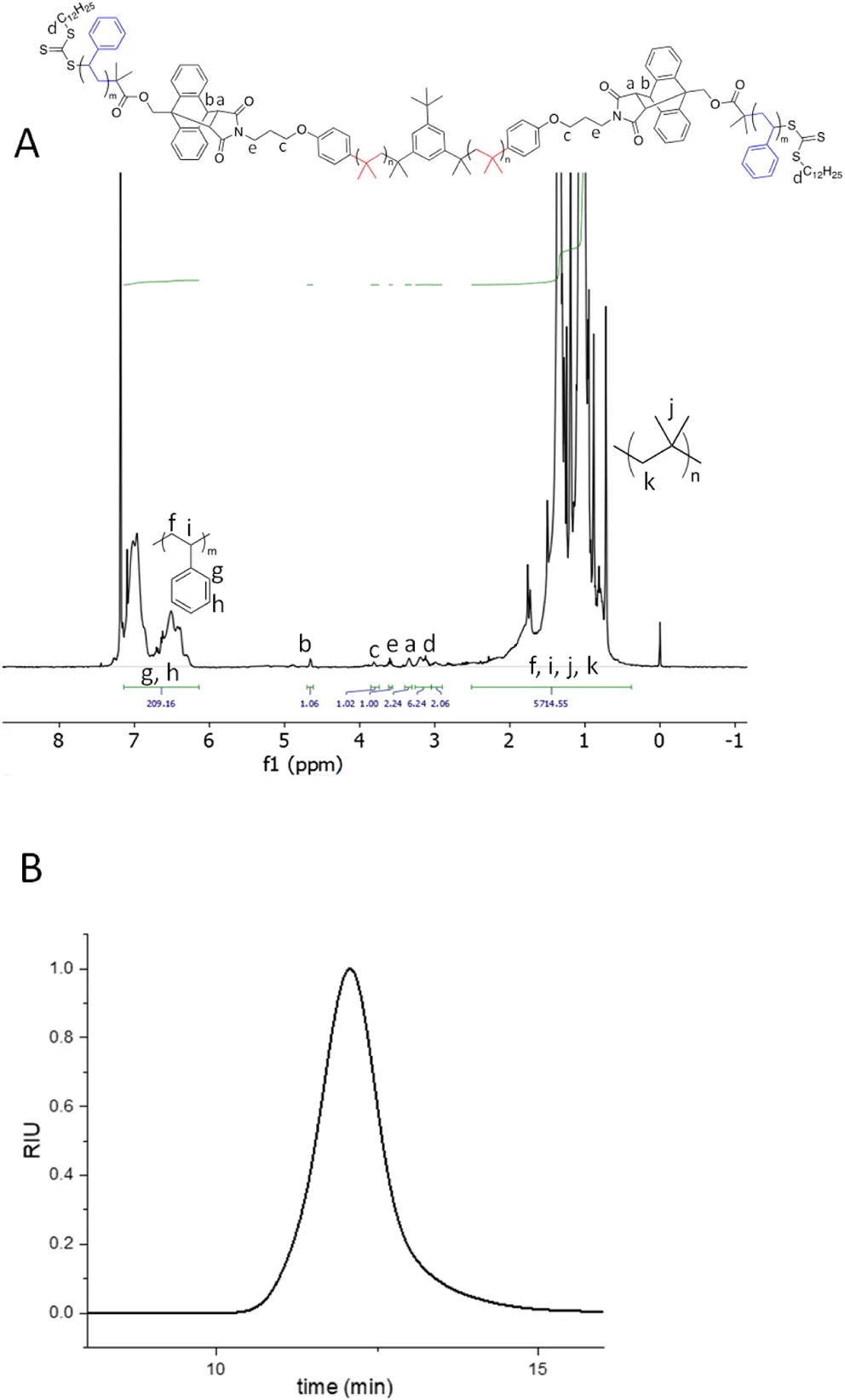
A) ^1^H NMR spectrum of PS_13_-M-PIB_30_-M-PS_13_ showing bridgehead protons from the mechanophore and quencher protons along with the backbone PIB and PS proton peaks. B) GPC trace showing a monomodal distribution of PS_17_-M-PIB_45_-M-PS_17_ confirming controlled polymerization.

Motivated by the results of a series of DFT calculations (see Section 3.3), a diblock copolymer containing no benzylic carbon and no mechanophore was synthesized to investigate the effects of low enthalpy linkages on chain scission. This allowed comparison of the diblock scission behavior with that of the triblocks containing the low enthalpy links. LCP with a monofunctional TMPCl initiator was employed to yield a block copolymer with a 110 kg/mol molecular weight containing a 54 kg/mol PIB block and a 55 kg/mol PS block (PIB:PS of 50:50) with a *Đ* of 1.32. The GPC trace of the block copolymer is shown in Figure S9.

### 3.2 Sonication

Pulsed ultrasound was used to evaluate chain scission of the block copolymers, following the procedure described in the experimental section. Dilute solutions in THF (a good solvent for both blocks of the copolymers) were sonicated, and aliquots were taken as a function of time. For each aliquot, GPC was performed to determine molecular weight and *Đ*, and mechanophore activation was determined by fluorescence spectroscopy.

#### 3.2.1 Chain Scission in PS–PIB–PS Triblock Copolymers without Mechanophore

Figure 4A shows an overlay of GPC plots of PS_30_–PIB_45_–PS_30_ as a function of sonication time. Monomodal peaks are observed as molecular weight decreases accompanied by a decrease in *Đ*, indicating that random scission is occurring as opposed to cleavage at one particular point on the chain.^56^ Similar monomodal degradation traces are observed for the other triblocks, shown in Figures S10-S12. This contrasts with the degradation behavior typically observed for homopolymers, where the chain cleaves at the center point, resulting in bimodal degradation curves with the molecular weight of the newly formed species equivalent to half that of the starting material.^57^ Such bimodal degradation patterns are attributed to the symmetric tension profile in homopolymers, which creates a maximum stress concentration at the midpoint,^57^ and are observed in the sonication of a PIB 45 kg/mol homopolymer (Figure 4B) and 110 kg/mol PIB and PS homopolymers (Figures S13 and S14). It is likely that the very different behavior observed in the triblock system results from the differences in force transduction throughout the chain induced by the presence of the two chemically distinct blocks.

**Figure 4:**
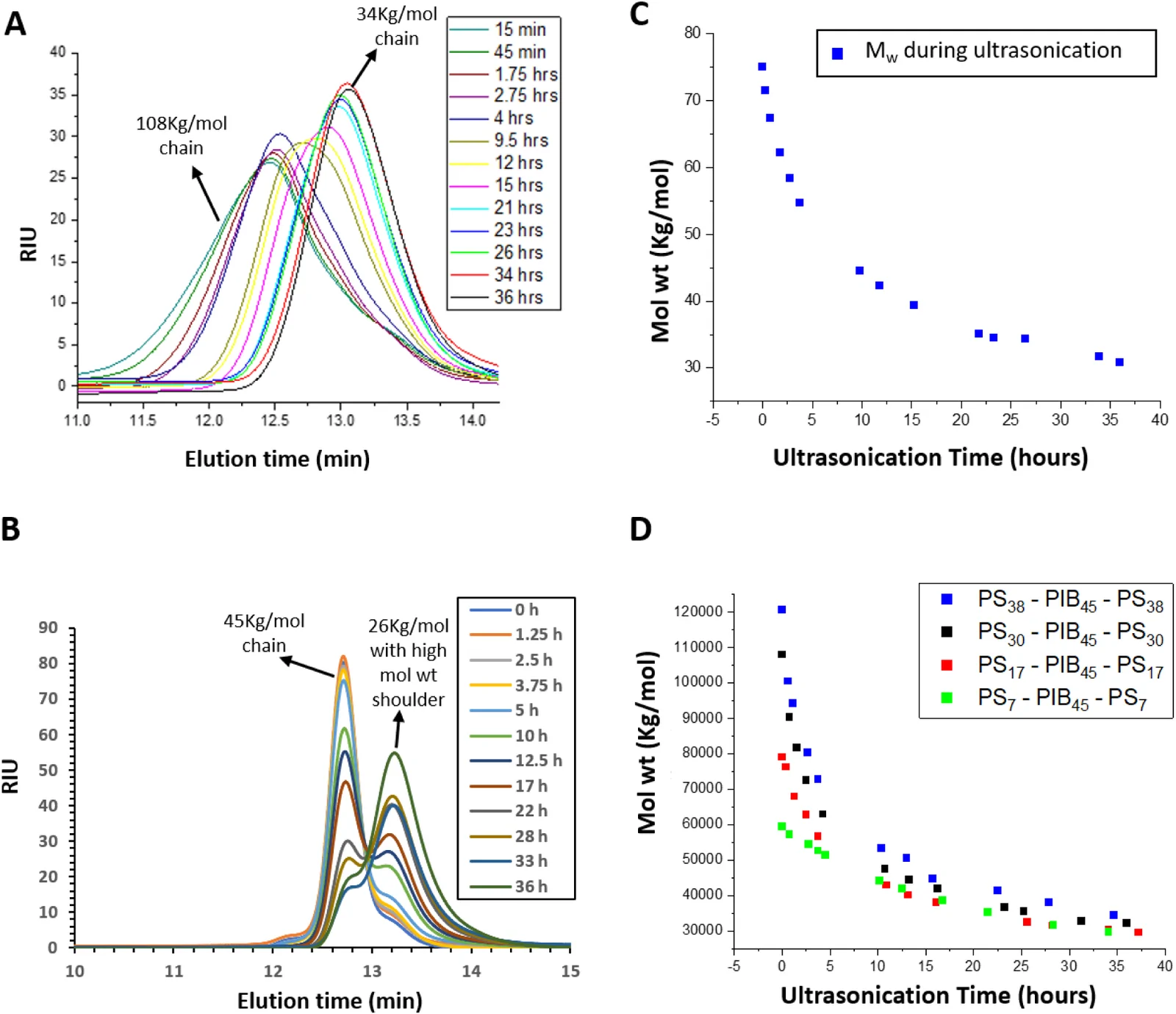
Chain scission behavior upon ultrasonication: A) GPC traces as a function of sonication time for PS_30_–PIB_45_–PS_30_ showing monomodal traces, B) GPC traces of PIB_45_ homopolymer showing bimodal degradation, C) *M*_w_ as a function of time for PS_30_–PIB_45_–PS_30_ showing exponential decay degradation, D) exponential decay degradation profiles for all four PIB_45_ triblocks.

A reduction in molecular weight is observed for the triblock copolymers, with a decrease following an exponential decay as described in eq 1, plateauing after approximately 30 h for all triblocks (Figure 4C for PS_30_–PIB_45_–PS_30_, Figure 4D for all 4 copolymers). A similar exponential decay is observed in *Đ*, as shown in Figure S15. It is interesting to note that while the exponential decay constant is higher for the higher molecular weight triblocks, all triblocks degrade to a plateau *M*_w_ of around 30 kg/mol after 36 h of sonication, which is lower than the PIB midblock *M*_w_ of 45 kg/mol, indicating that scission is not limited to just one block or to the junctions between blocks, but rather is distributed randomly across the contour of the chain.^56^ The monomodal, random degradation profiles indicate that the presence of junctions plays an important role in chain scission in block copolymers, as predicted by the computational studies done by Zhang et al.,^35^ and there is no preferential cleavage of one particular block. The PIB and PS homopolymers show a similar ultimate *M*_w_ (around 30 kg/mol), but this results from specific cleavage at the center of the chain (Figure 4B).

The Baramboim kinetic equation (shown in eq 1) is widely used for studying degradation kinetics in homopolymers and block copolymers.^58–60^

The ultrasonication data were fitted to eq 1:

1 $$M_{w}\left(t\right)=M_{\lim}+\left(M_{w,0}-M_{\lim}\right)e^{-kt}$$

where

*M*_w_(*t*) = molecular weight at time *t*.

*M*_lim_ = limiting molecular weight at the plateau after sonication.

*M*_w,0_ = initial molecular weight at *t* = 0 before sonication.

*k* = rate of chain scission.

*t* = time at which an aliquot was taken during ultrasonication.

Figure 5 shows model parameters and curves for the four triblocks, indicating good fits with eq 1. The increase in *k* with increasing *M*_w,0_ can be explained by theories of coil stretching under elongational and shear flow.^61–63^ The high initial chain length makes the triblocks more susceptible to scission due to greater coil stretching under cavitation-induced elongational and shear forces, while the lower *M*_w,0_ samples have lower induced stretching and higher resistance to cleavage. The higher *M*_w_ chains have higher contour lengths, allowing bubble collapse over the length of the chain, leading to scission. A steep reduction in *M*_w_ in the initial 10 h of scission is observed when long chains are still present in solution. The critical strain rate required to cleave a chain is inversely proportional to the molecular weight of the chain (E_c_ ∝ *M*_w_^–x^, where x = 1.7 to 2).^11^ Thus for a given strain rate during sonication, greater cleavage is expected when higher molecular weight chains are present. The triblocks converged to the same *M*_lim_ range (25–35 kg/mol) irrespective of the *M*_w,0_, suggesting that the sonication conditions have a more pronounced effect on ultimate chain scission than the triblock composition and block length.

**Figure 5:**
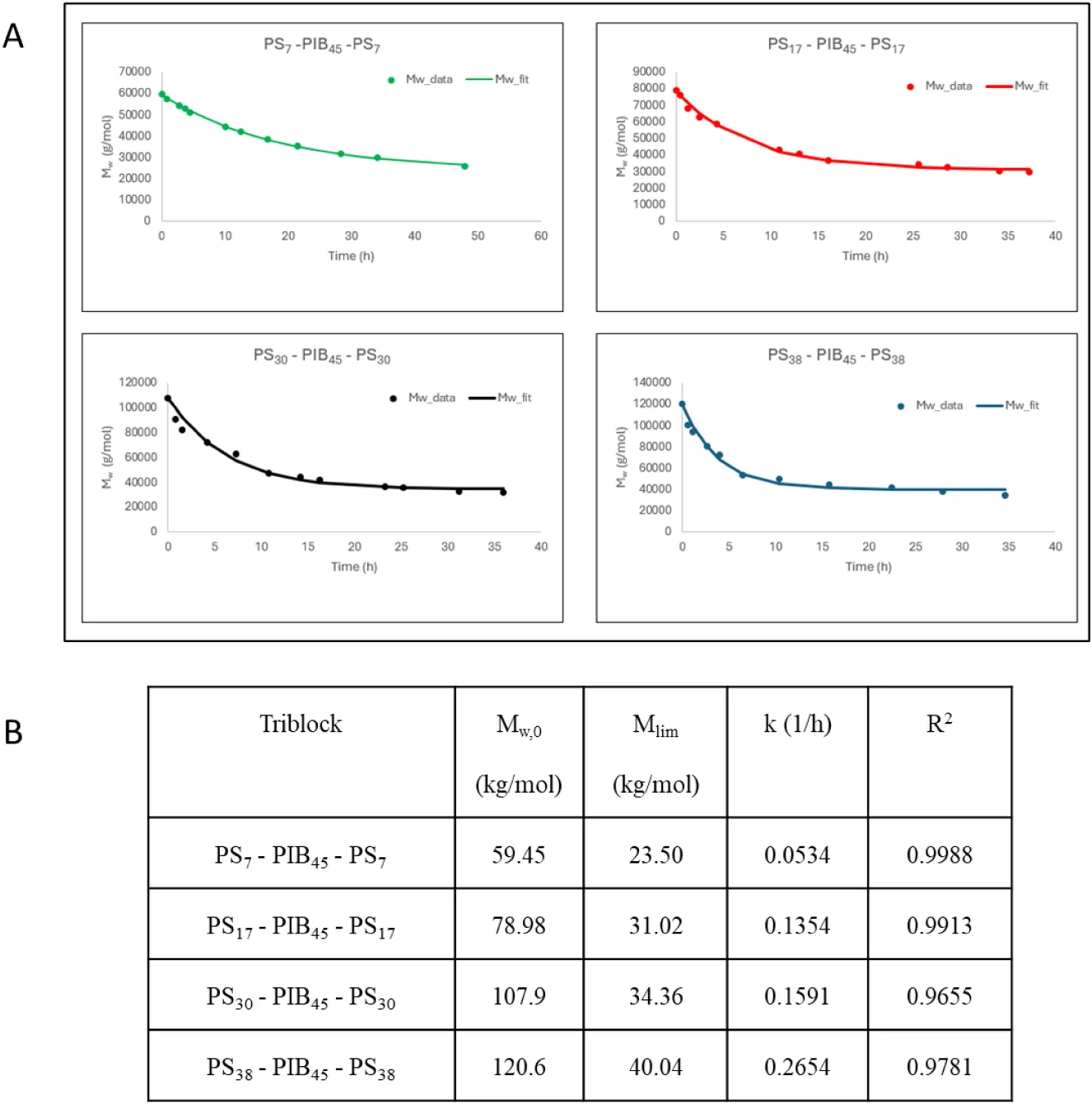
A) Baramboim model fit for the molecular weight degradation curve in triblocks, demonstrating good agreement of the data with the models. B) Baramboim model fit parameters for all triblocks without mechanophores.

#### 3.2.2 Chain Scission in Mechanophore-Containing Block Copolymers

Fluorescence spectroscopy analysis, GPC analysis, and molecular weight degradation profiles as a function of sonication time of the mechanophore-containing triblock copolymers with PIB_30_ center blocks are shown in Figure 6A, Figure 6B, Figure 6C, and Figures S16–S18; the corresponding data for those with PIB_45_ center blocks are shown in Figures S19–S20. Monomodal GPC traces and degradation patterns with *M*_lim_ of ∼30 kg/mol are observed for all samples, similar to those observed for the triblocks without mechanophores (Figure S21). The Baramboim model fits the data well, and the rate of scission increases with increasing *M*_w,0_ as observed for the control triblocks (Figure S22 and Table S2). Thus, incorporation of a mechanophore at the interface does not affect chain scission in the triblock due to the similarity in the GPC degradation profiles and the rate constants during degradation predicted by the Baramboim model. Fluorescence spectroscopy overlay curves with increasing sonication time show a continuous increase in fluorescence intensity (Figure 6A), resulting from the generation of free anthracene units upon mechanophore cleavage via a retro-Diels–Alder reaction. Overall mechanophore activation is very low, less than 5%, indicating that chain scission does not occur primarily at the interface of the blocks.^26,30^ A plot of normalized *M*_w_ and normalized fluorescence intensity as a function of sonication time for PS_22_-M-PIB_30_-M-PS_22_ (Figure S23) shows that the rates are decoupled. In the initial stages (<10 h sonication), the rate of *M*_w_ decrease is greater than the rate of increase in fluorescence intensity. In the later stages (>10 h sonication), the rate of fluorescence increase is high, while the rate *M*_w_ decrease begins to plateau. Thus, the combined GPC and fluorescence spectroscopy analysis demonstrates that although mechanophores are activated continuously during ultrasonication, the chains are not broken preferentially at the mechanophore linkage, nor at the center of the chain, as evidenced by the absence of bimodal GPC curves and peaks corresponding to half the molecular weight indicating chain scission at the center, and the scission takes place in a nonselective manner.

**Figure 6:**
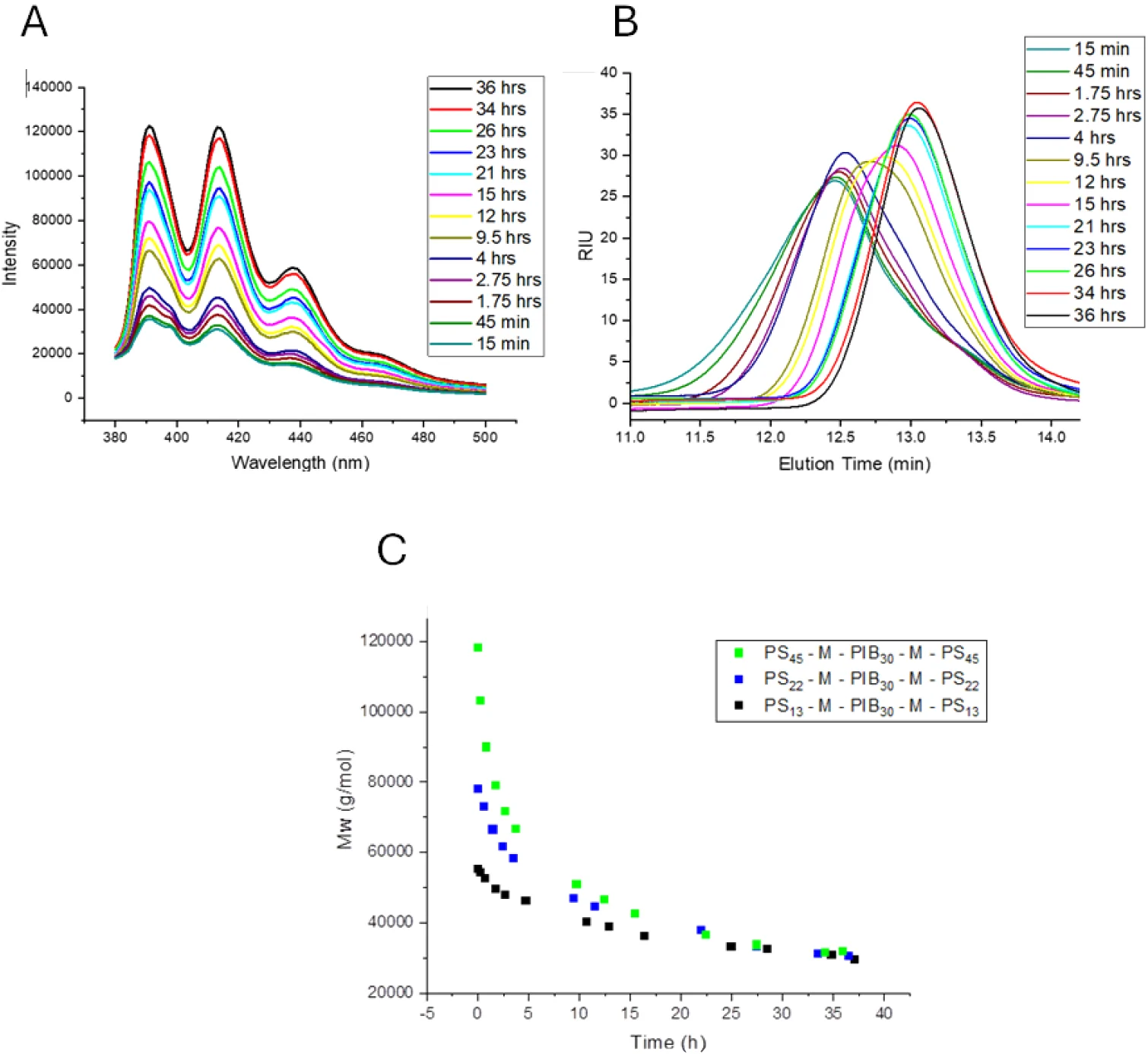
A) fluorescence intensity with increasing sonication time for PS_22_-M-PIB_30_-M-PS_22_, indicating continuous mechanophore cleavage, B) monomodal GPC overlays measuring *M*_w_ degradation with increasing sonication time, indicating nonselective chain scission for PS_22_-M-PIB_30_-M-PS_22_, and C) comparative degradation profiles for the 3 block copolymers with 30 kg/mol PIB center blocks, showing exponential decay degradation with a plateau MW of ∼30 kg/mol.

Mechanophore activation was evaluated using fluorescence spectroscopy of aliquots taken during ultrasonication, as described in the experimental section. Each sample was evaluated in triplicate. Average mechanophore activation as a function of sonication time for the PIB_30_ and PIB_45_ triblocks is shown in Figure S24A,B. For both copolymer systems, the rate and ultimate level of mechanophore activation increase with increasing *M*_w,0_. For triblocks of similar overall *M*_w,0_ but lower PS chain lengths, ultimate mechanophore activation is reduced. PS_45_-M-PIB_30_-M-PS_45_ and PS_37_-M-PIB_45_-M-PS_37_ both have *M*_w,0_ of 120 kg/mol, but their ultimate activations are 4.2% ± 0.1% and 3.8% ± 0.1%, respectively. Similarly, the 80 kg/mol PS_22_-M-PIB_30_-M-PS_22_ shows higher mechanophore activation than its counterpart PS_17_-M-PIB_45_-M- PS_17_ (2.5 ± 0.09% vs 1.0 ± 0.09%). Thus, higher PS phase content in the block copolymer leads to higher mechanophore activation at the block interface. This may be attributed to differences in chain conformation, with a more extended conformation exhibited for copolymers with higher PS content. The higher mechanophore activation found in the 120 kg/mol triblocks in comparison to the 80 kg/mol triblocks may also be attributed to the higher molecular weight of their PS chain ends: >35 kg/mol and 15 kg/mol, respectively. The 15 kg/mol chain end is close to the 18 kg/mol PS cutoff molecular weight, below which scission does not occur.^64,65^ We previously observed similar trends in PIB–PS graft copolymers, wherein an increase in the mechanophore activation was observed when the molecular weight of PS chains was higher than 13.8 kg/mol.^32^

To evaluate the effect of dispersity, the two lower *Đ* copolymers (Figure S8) were subjected to sonication and compared to their higher *Đ* analogues. Average mechanophore activation for the low and high *Đ* samples is within ±1% (Figure S25), and the curves for molecular weight as a function of sonication time overlap (Figure S26). As the *Đ* of our copolymers is lower than 1.65, the relatively small differences in the low and high tails of the molecular weight distribution curves do not greatly affect chain scission profiles. The degradation profiles for all samples follow similar trajectories, with chain scission dictated by the length of the chain, in good agreement with the Baramboim kinetic model.

### 3.3 Effect of Absence of Low Enthalpy Bonds on Chain Scission

Density functional theory (DFT) calculations were performed to calculate the enthalpy of reaction (Δ_r_ *H*°) values for several chemical moieties of interest along the triblock copolymer. These calculations confirmed the low Δ_r_ *H*° (134 kJ/mol) of the mechanophore relative to the rest of the carbon backbone. The calculations also indicated that a pair of benzylic quaternary carbons, one located near the polymer chain center at the bDCC initiator site, and the other at the point of addition of the PIB quencher, each had reduced Δ_r_ *H*° values of 274 and 273 kJ/mol, respectively. This constitutes an approximately 24–37 kJ/mol reduction relative to other areas of the polymer backbone, as explained in detail in the experimental section and shown in Figures S27–S32 and Table S3.

In order to evaluate the effect of the low enthalpy linkages of the triblock on chain scission, a PIB–PS diblock copolymer without the mechanophore at the interface and without the benzylic quaternary carbons was synthesized as described in the experimental section. Ultrasonication of this sample reveals that the degradation behavior of the diblock copolymer, in the absence of the low enthalpy linkages, is similar to that of the triblock copolymers. GPC analysis shows monomodal degradation curves for the diblock (Figure S33A), and similar chain scission trajectories are observed for the diblock in comparison to a mechanophore-containing triblock of similar molecular weight (Figure S33B). These findings indicate that the lowest-enthalpy bond does not direct the forces of cleavage on the chain in our system. The similar nature of chain scission at a particular molecular weight of the triblock, irrespective of the presence of mechanophores, indicates that the physical forces instrumental in concentrating the stress on the chain play a greater role in determining chain scission than the bond enthalpy. Thus, for the first time in triblock copolymers, we report experimental validation of the computational results by Zhang et al. and Chongvimansin et al., which suggest the predominant effect of physical factors (conformations and plasticity) on the work of scission over bond energies.^35,36^ Recent MD simulation studies have shown that chain conformations adopted by single chains in dilute solutions of good solvents, such as random coil or spherical conformations, play an important role in structural preorganization, thereby leading to overstretching and subsequent chain scission in an off-center position.^35,36,66^

### 3.4 Effect of Solvent Type on Chain Scission

Solvent mixtures with varying levels of compatibility with the two blocks were prepared to evaluate the effect of the solvent environment on the chain scission. THF is a good solvent for both blocks, methanol is a poor solvent for both blocks, hexanes is a good solvent for PIB but a poor solvent for PS, and acetone is a good solvent for PS but a poor solvent for PIB (Table S4). Solvent mixtures of the good solvent, THF, with the poor solvents were prepared at a 70:30 ratio to prevent precipitation of the block copolymer upon the addition of the poor solvent. Ultrasonication of PS_30_–PIB_45_–PS_30_ in THF, THF:hexanes (70:30), THF:acetone (70:30), and THF:methanol (70:30) was performed to evaluate chain scission under full extension (THF), partial coiling (hexanes, acetone), and fully coiled (methanol) states.

As seen in the molecular weight degradation profile in Figure S34A and the Baramboim kinetic equation fit parameters in Table S5, *M*_lim_ was highest for methanol (56 kg/mol), followed by acetone and hexanes (40 kg/mol and 37 kg/mol), and lowest for THF (32 kg/mol). *M*_lim_ is governed by chain coiling, which lowers the maximum extensional force transmitted to the polymer backbone during ultrasonication. Following this logic, we would expect the initial rate of scission to follow the inverse trend, with the extended chains in THF showing the highest rate and the coiled chains in methanol showing the lowest rate. While the initial rate of scission was found to be highest in THF (0.25 h^–1^), it was intermediate in methanol (0.17 h^–1^), and lowest in hexanes (0.14 h^–1^) and acetone (0.12 h^–1^). We propose that this may be related in part to the lower vapor pressure of methanol in comparison to that of acetone and hexanes, which allows stronger bubble collapse with higher drag forces on the chain as compared to solvents with higher vapor pressure, which display acoustic cushioning leading to lower intensity of cavitation forces. Thus, the geometric threshold, which determines the *M*_lim_, and the cavitation energetics, which determine *k*, can be modulated by the solvent used in ultrasonication. This phenomenon has been reported in a few cases in homopolymers and self-assembled systems,^62,63,67–69^ but to our knowledge it has not yet been reported for triblock copolymers.

The same solvent study was performed for the mechanophore-containing triblock, PS_37_-M-PIB_45_-M-PS_37_, and mechanophore activation was determined by fluorescence spectroscopy, as shown in Figure S34B. *M*_lim_ values are very similar to those obtained for the triblocks without mechanophores. Mechanophore activation is low in all solvents tested (<5%) and decreases in solvents of poorer quality. These findings provide further evidence that physical factors such as chain conformation and vapor pressure play a larger role in scission than do the chemical effects of standard bond enthalpies. In a good solvent, chains were more expanded and isolated, allowing cavitation-induced flows to impose large forces across the junction, producing both rapid scission and measurable mechanophore activation. In the presence of poor solvents, coil compaction, screening, and acoustic damping reduced the per-chain stress, yielding slower scission and reduced activation. The mechanophore activation was reduced significantly below 2% when triblocks were ultrasonicated in poor solvents, thereby mimicking the mechanophore activation of short chains (PS_13_-M-PIB_30_-M-PS_13_) in good solvents. Thus, we have demonstrated that by tuning solvent quality, the extent of both chain scission and junction mechanophore activation can be predictably modulated, offering a mechanistic framework for designing block copolymer mechanochemical responses under ultrasound. Similar findings were reported in computational studies, wherein solvent quality was found to affect the contour length of structures, the alignment of polymeric chains in solution, and the drag force experienced by the chains, all leading to differences in mechanophore activation and the site of scission.^35,36,66^

## 4 Conclusion

We have demonstrated the use of a grafting-from approach to synthesize PS–PIB–PS triblock copolymers with anthracene–maleimide mechanophores at the block interfaces. The synthesis of a well-defined library of PS–PIB–PS triblock copolymers, with systematic variations in molecular weight, block composition, dispersity, and mechanophore incorporation, allowed for the evaluation of the effects of molecular structure on chain scission in thermoplastic elastomers. Solution-state ultrasonication was employed to create elongational and shear flow under high strain rate acoustic cavitation to induce scission in polymer solutions, and solvent mixtures of varying compatibility with the individual blocks were evaluated. The synthesis of diblock copolymers without the lowest enthalpy bonds identified by DFT analysis, with the mechanophore having a reaction enthalpy of 134 kJ/mol and the quaternary benzylic units having bond enthalpies of ∼273 kJ/mol, allowed for interrogation of the site of chain cleavage. GPC and fluorescence spectroscopy analysis as a function of sonication time reveal monomodal degradation profiles and low levels of mechanophore activation, suggesting that chain scission is not selective, and does not occur at the block interface, at the lowest enthalpy bond, or at the chain center in the triblock copolymers. Mechanophore activation was the highest, at 4.2%, for the triblock with the highest PS content, and was very low, only 0.5%, when the molecular weight of PS fell below 13.8 kg/mol, the cutoff degradation molecular weight of PS. Hence the stress concentrated at the interface can be tuned depending on the length and composition of the blocks within the copolymer chain.

Variations in the physical environment during ultrasonication led to appreciable changes in chain scission and mechanophore activation. In a good solvent, the limiting molecular weight of triblocks of all molecular weights and compositions was ∼30 kg/mol. The introduction of a poor solvent/good solvent mixture, in which chains are expected to exhibit a coiled rather than an extended conformation, increased *M*_lim_ to 50 kg/mol and reduced mechanophore activation to 1.9%. The rate of chain scission was found to depend on physical properties such as the viscosity and vapor pressure of the solvent.

In this work, we established that covalent bond rupture in triblock copolymers under high-strain-rate ultrasonication is dictated by chain conformation, plasticity, and the physical properties of the sonication medium rather than the bond enthalpies of the chemical linkages in the chain. We have thereby addressed a current knowledge gap in polymer mechanochemistry pertaining to the driving force responsible for the chain scission of block copolymers during acoustic cavitation. Our findings are consistent with computational studies reported in the literature and establish an experimental baseline for understanding the effects of factors such as chain architecture, conformation, and sites of stress concentration on the solution-state response of block copolymers under shear and elongational forces.

## Supplementary Material



## Notes

Fluorescence spectroscopy calibration curve with PS-Anth; mechanophore activation calculated from fluorescence intensity of free anthracene; fluorescence spectroscopy to monitor Diels–Alder reaction; GPC plots of: control triblocks without mechanophore, mechanophore-containing triblocks, low and high *Đ* triblock copolymers, PIB–PS diblock copolymer; GPC overlay plots as a function of sonication time for: control triblocks without mechanophore, PIB and PS homopolymers, mechanophore-containing triblock copolymers; ^1^H NMR of triblock copolymer; reaction scheme and ^1^H NMR of anthracene-functional RAFT agent; plot of *Đ* as a function of sonication time for triblock copolymer; plots of molecular weight as a function of sonication time for control triblocks, mechanophore-containing triblocks, low and high *Đ* block copolymers, diblock copolymer without low enthalpy bonds, triblock copolymers in solvents of different strength; plots of Baramboim model fits with equation fit parameters for mechanophore-containing triblock copolymers; normalized *M*_w_ and normalized fluorescence intensity plotted as a function of sonication time for triblock copolymer; plots of mechanophore activation as a function of sonication time for: triblock copolymers, low and high *Đ* block copolymers; structures and equations used for standard reaction enthalpy calculations; tables of computationally calculated enthalpies of reaction, solvent quality, Baramboim equation fit parameters for triblock copolymer in solvents of different quality (PDF)
