# Supplementary material for: Mechanisms of Chain Scission under High Strain Rate Sonication in PS–PIB–PS Triblock Copolymers
Source: ACS Appl Polym Mater. 2026 Aug 17;8(16):14086–99. doi: 10.1021/acsapm.6c02287 (PMC13532198; doi:10.1021/acsapm.6c02287)
Supplement: Supplementary file 1 [file ap6c02287_si_001.pdf]

Supporting Information

Mechanisms of Chain Scission under High Strain Rate Sonication in PS-PIB-PS Triblock Copolymers

Parth K. Vagholkar[1], Alexander J. Rosario[1], John F. Searles[1], Lisa K. Kemp[1], Yoan C. Simon[2], Robson F. Storey[1], Boran Ma[1], Dane N. Wedgeworth[3], Travis Thornell[3], Sarah E. Morgan[1,*]

[1]School of Polymer Science and Engineering, University of Southern Mississippi, Hattiesburg, Mississippi, 39406, USA

[2]School of Molecular Sciences, Arizona State University, Tempe, Arizona, 85287, USA

[3]Geotechnical and Structures Laboratory, Engineer Research and Development Center (ERDC), US Army Corps of Engineers (USACE), Vicksburg, Mississippi, 39180, USA

*E-mail: sarah.morgan@usm.edu

PS-Anth standards	
Concentration (mol/dm^3)	Intensity
1.00E-04	10713743
5.40E-05	5805000
1.08E-05	1065000
2.16E-06	321000
4.32E-07	88200
8.66E-08	33660
1.73E-08	10730
3.46E-09	7346
6.93E-10	5069.3
1.39E-10	5013.9

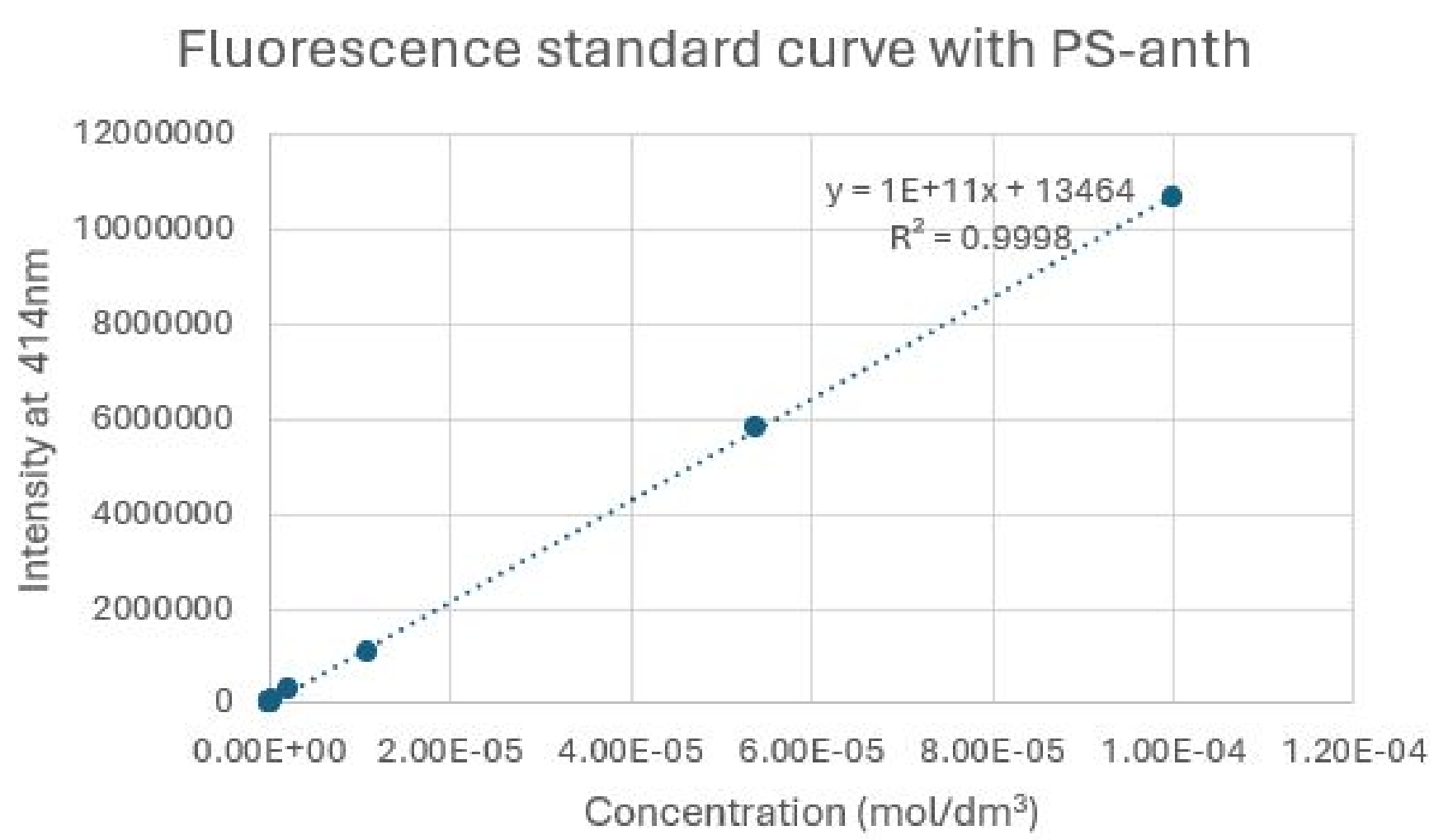

Figure S1: Fluorescence spectroscopy calibration curve using PS-Anth with varying concentrations in THF at λexc = 356 nm, λem = 414 nm.

Table S1: Intensity of free anthracene (at 414 nm) and mechanophore activation values after 72 hours of sonication.

Sample	Initial Mw (GPC) before sonication (Kg/mol)	[Anth] at 100% activation	Fluoresecence Intensity at 414nm at 100% activation	Fluoresecence Intensity at 414nm after 72 h	MECHANOPHORE ACTIVATION (%)
PS_{13} - M - PIB_{30} - M - PS_{13}	56	2.85E-06	6.00E+06	30019	0.5
PS_{22} - M - PIB_{30} - M - PS_{22}	75	2.14E-06	4.80E+06	120000	2.5
PS_{45} - M - PIB_{30} - M - PS_{45}	118	1.36E-06	3.34E+06	140000	4.2
PS_{17} - M - PIB_{45} - M - PS_{17}	79	2.03E-06	4.32E+06	43227	1
PS_{37} - M - PIB_{45} - M - PS_{37}	120	1.34E-06	3.53E+06	134000	3.8

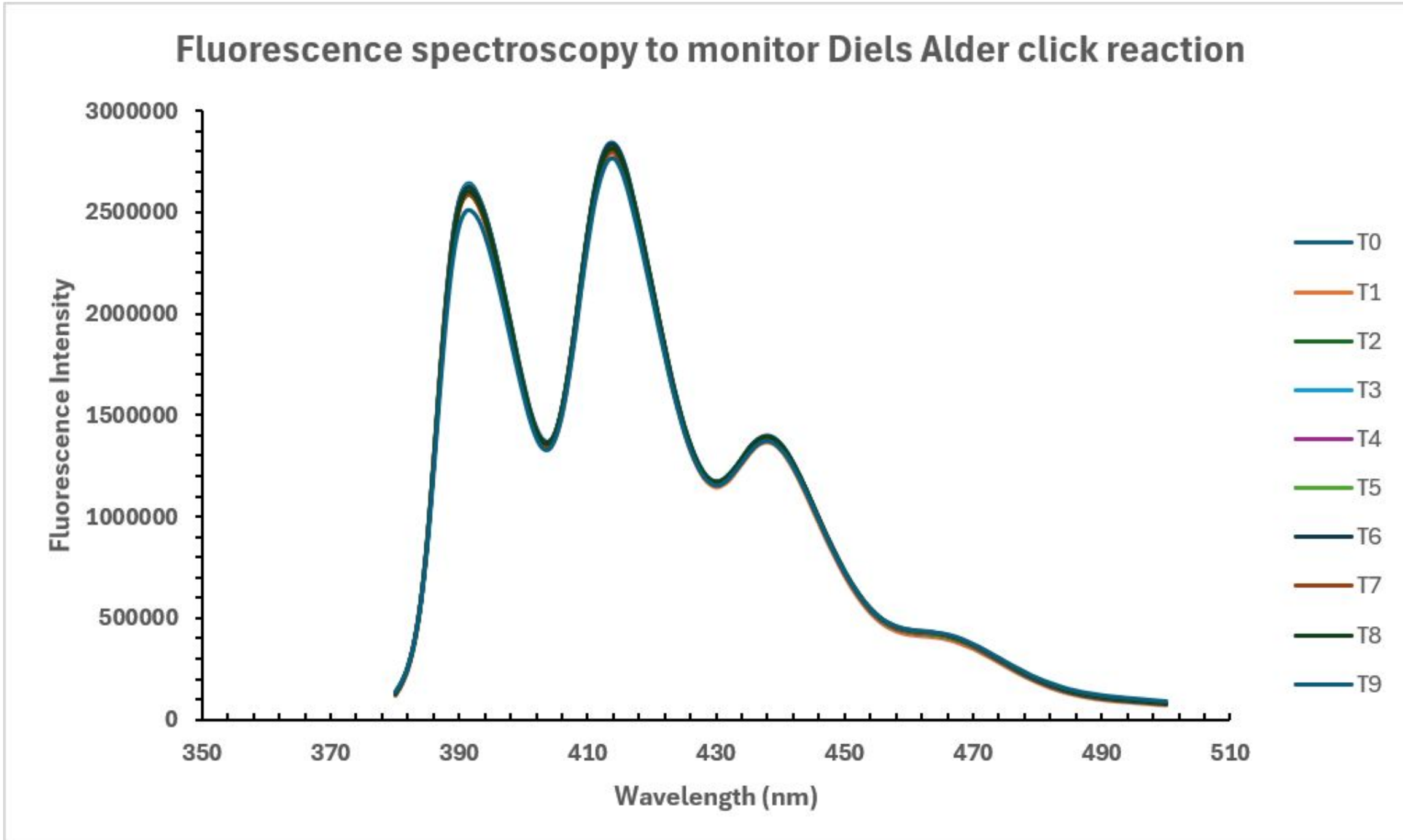

Figure S2: Fluorescence intensity of PIB-MI, PS-Anth solution aliquots taken during sonication for 3 days under under N_2 atmosphere at 5ºC. No appreciable reduction in anthracene fluorescence intensity is observed.

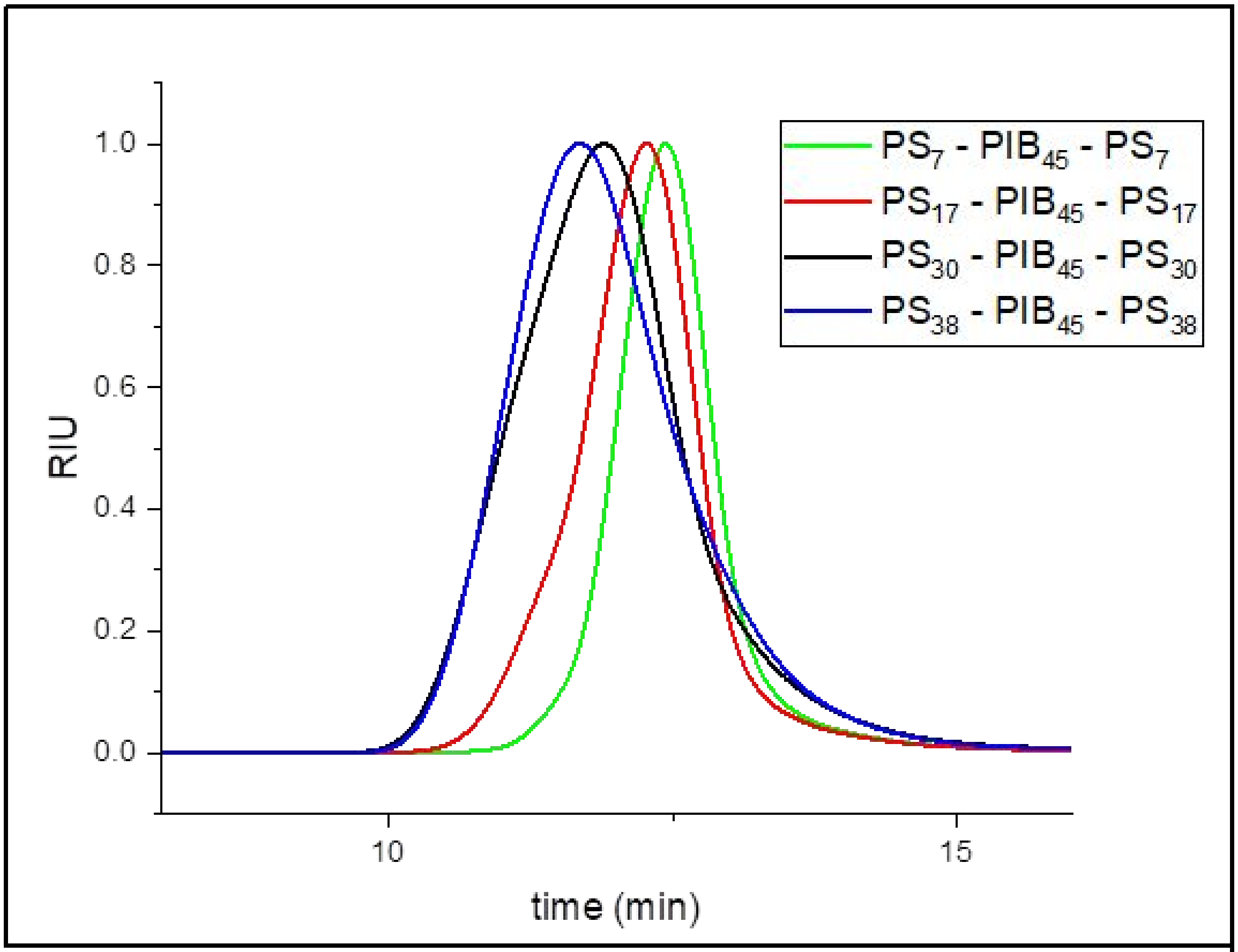

Figure S3: GPC overlay plots for the synthesized control triblocks with 45kg/mol PIB midblock and no mechanophore, showing monomodal distributions and increasing Mw with increasing PS block length.

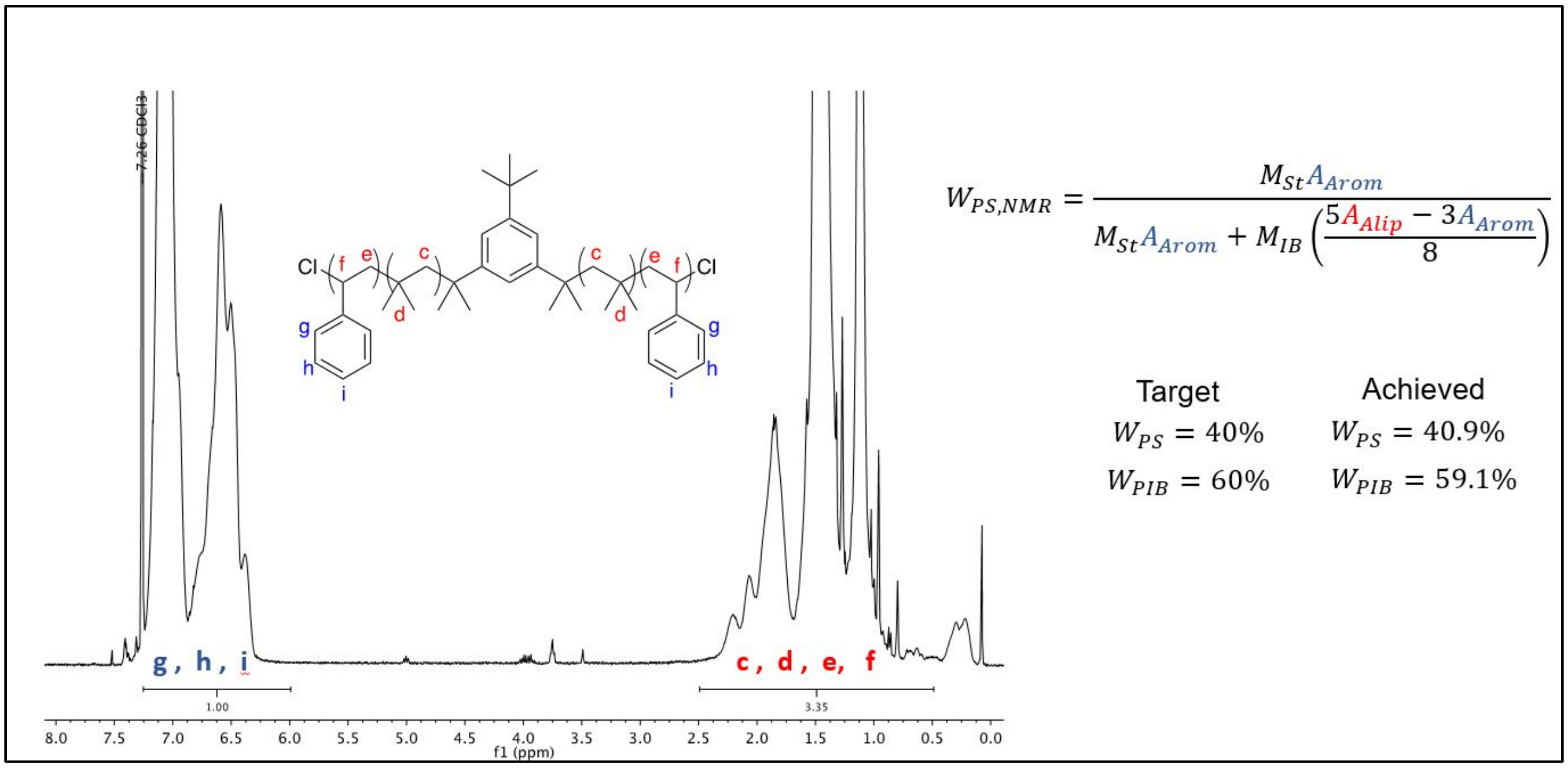

Figure S4: ^{1}H NMR spectrum of $PS_{17} – PIB_{45} – PS_{17}$ and method for calculating weight percent of PIB and PS in the block copolymer.

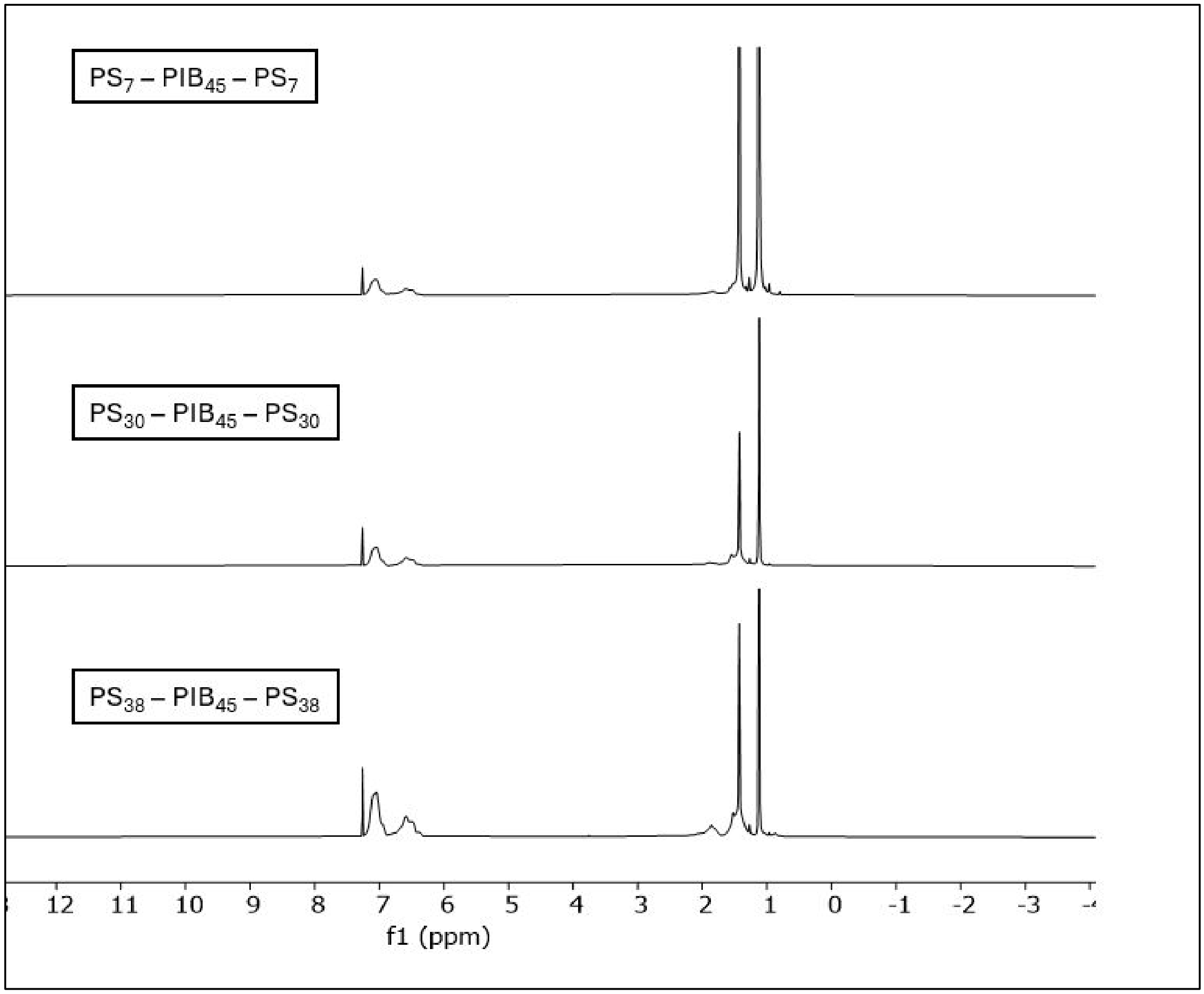

Figure S5: ^{1}H NMR stacked spectra of triblock copolymers with 45kg/mol PIB midblock

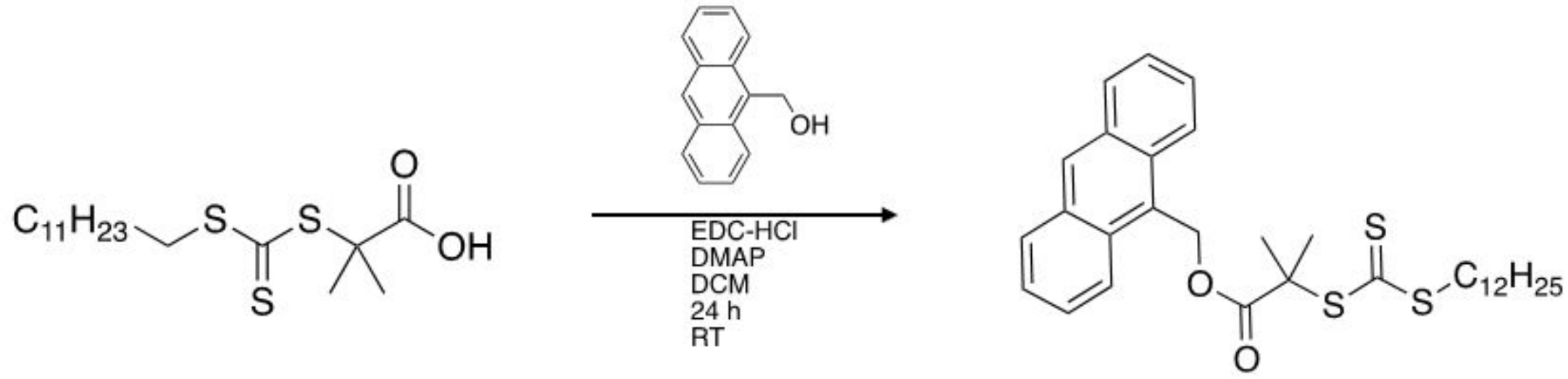

Scheme S1: Synthesis of anthracene-functional RAFT agent

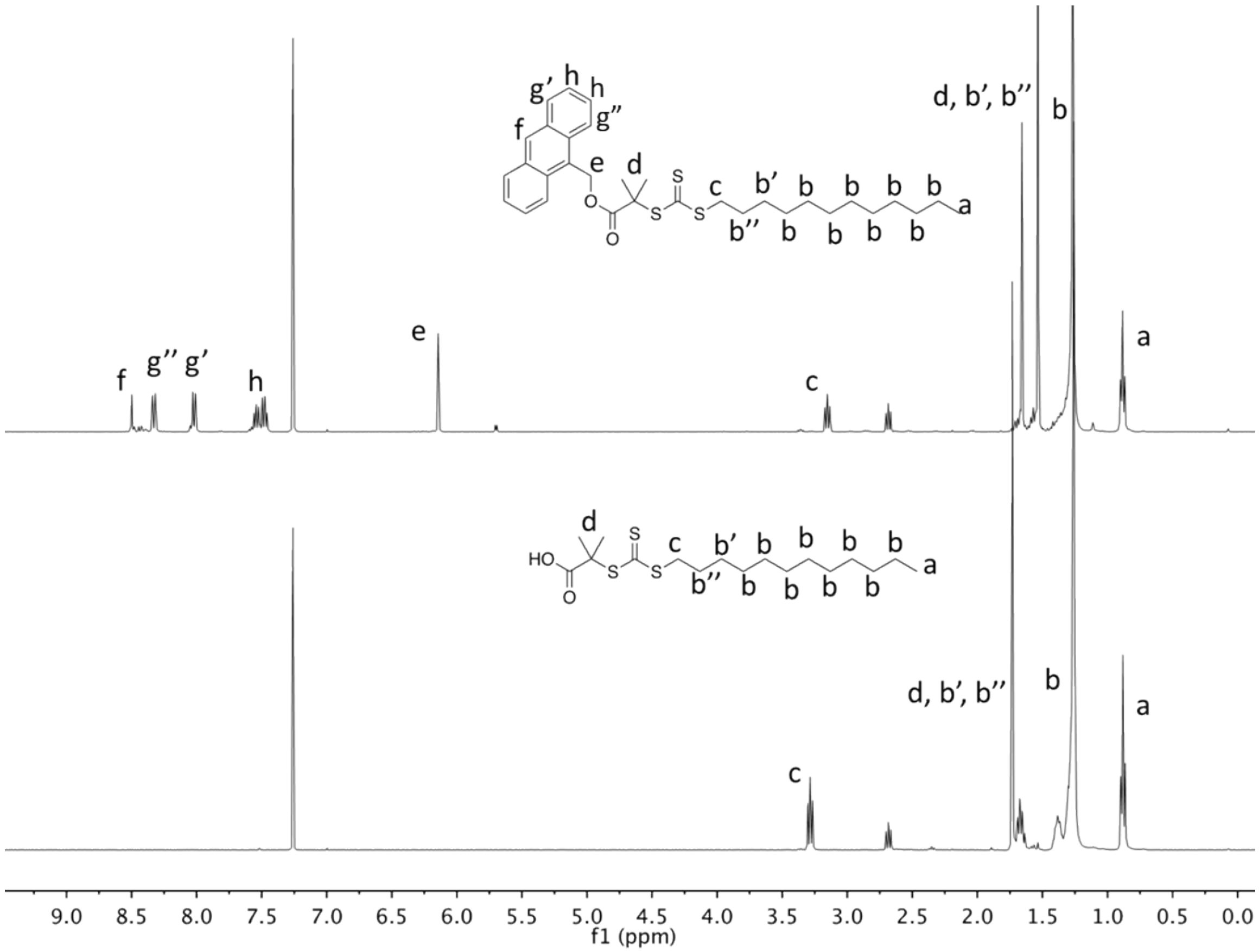

Figure S6: ^{1}H NMR spectra showing formation of anthracene functional RAFT agent (top) and neat RAFT agent (bottom).

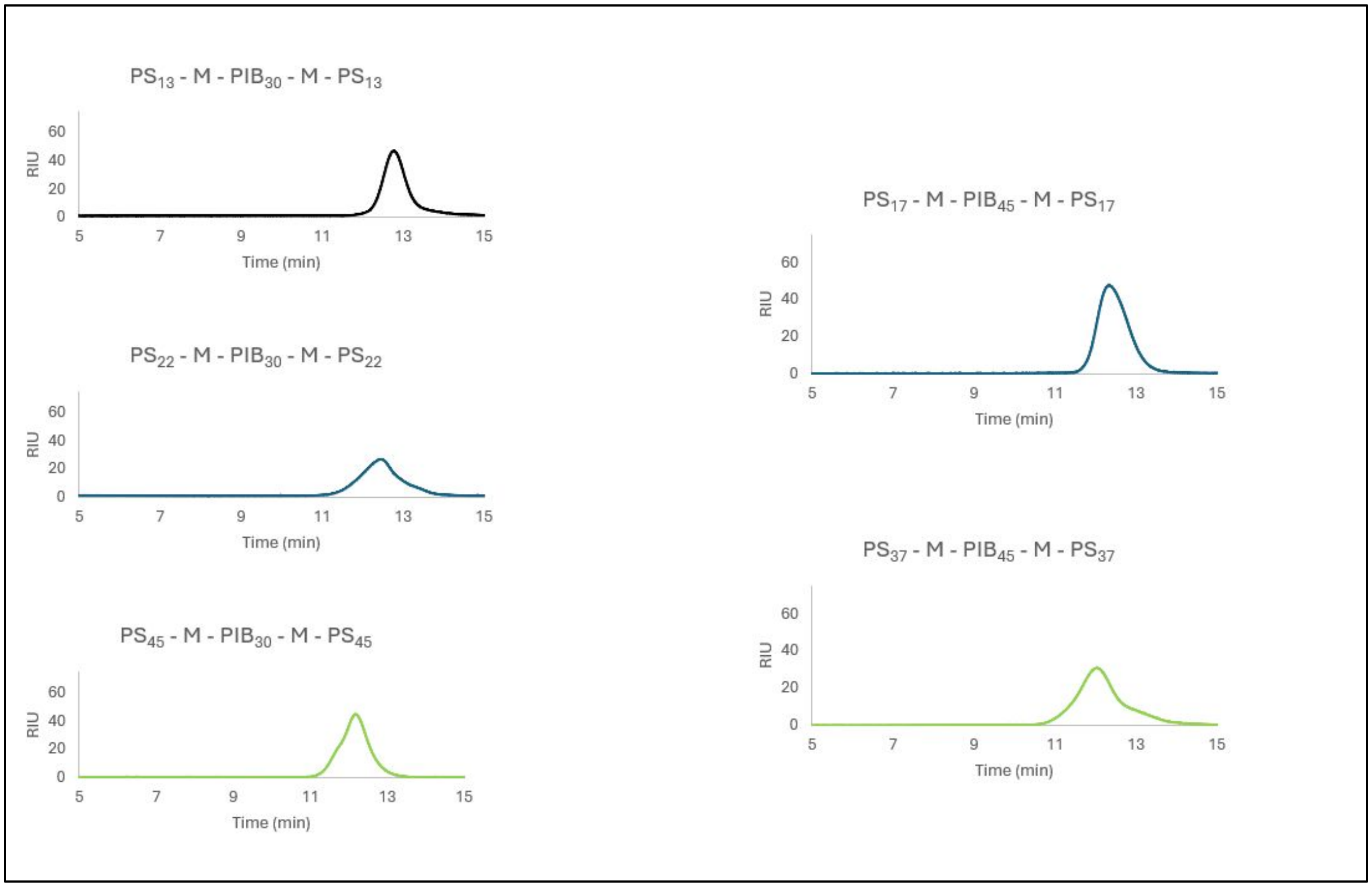

Figure S7: GPC traces of the synthesized mechanophore-containing triblocks with 30kg/mol and 45g/mol PIB midblock showing monomodal distributions and increasing Mw with increasing styrene content.

Triblock	Mol wt (Kg/mol)	Đ	%PS
PS_{37}-M-PIB_{45}-M-PS_{37}	120	1.61	71
*PS_{37}-M-PIB_{45}-M-PS_{37}	126	1.28	76
PS_{22}-M-PIB_{30}-M-PS_{22}	75	1.41	60
*PS_{22}-M-PIB_{30}-M-PS_{22}	78	1.27	60

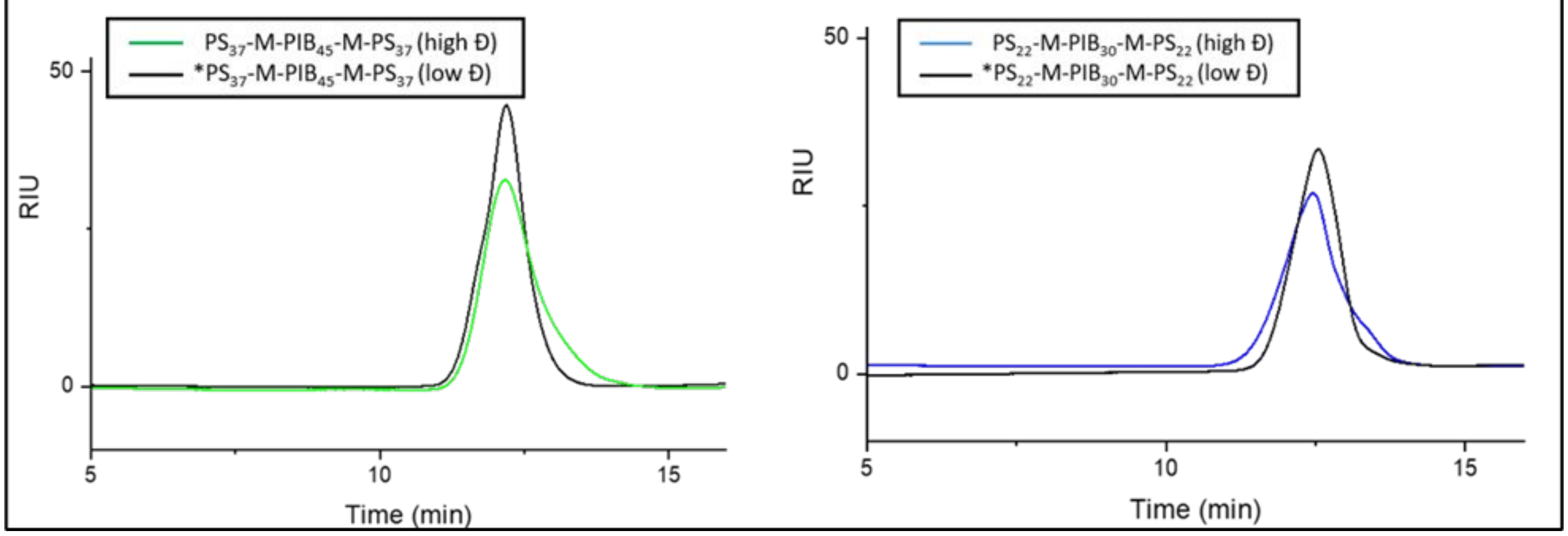

Figure S8: Mechanophore containing triblocks with narrow and broad dispersities

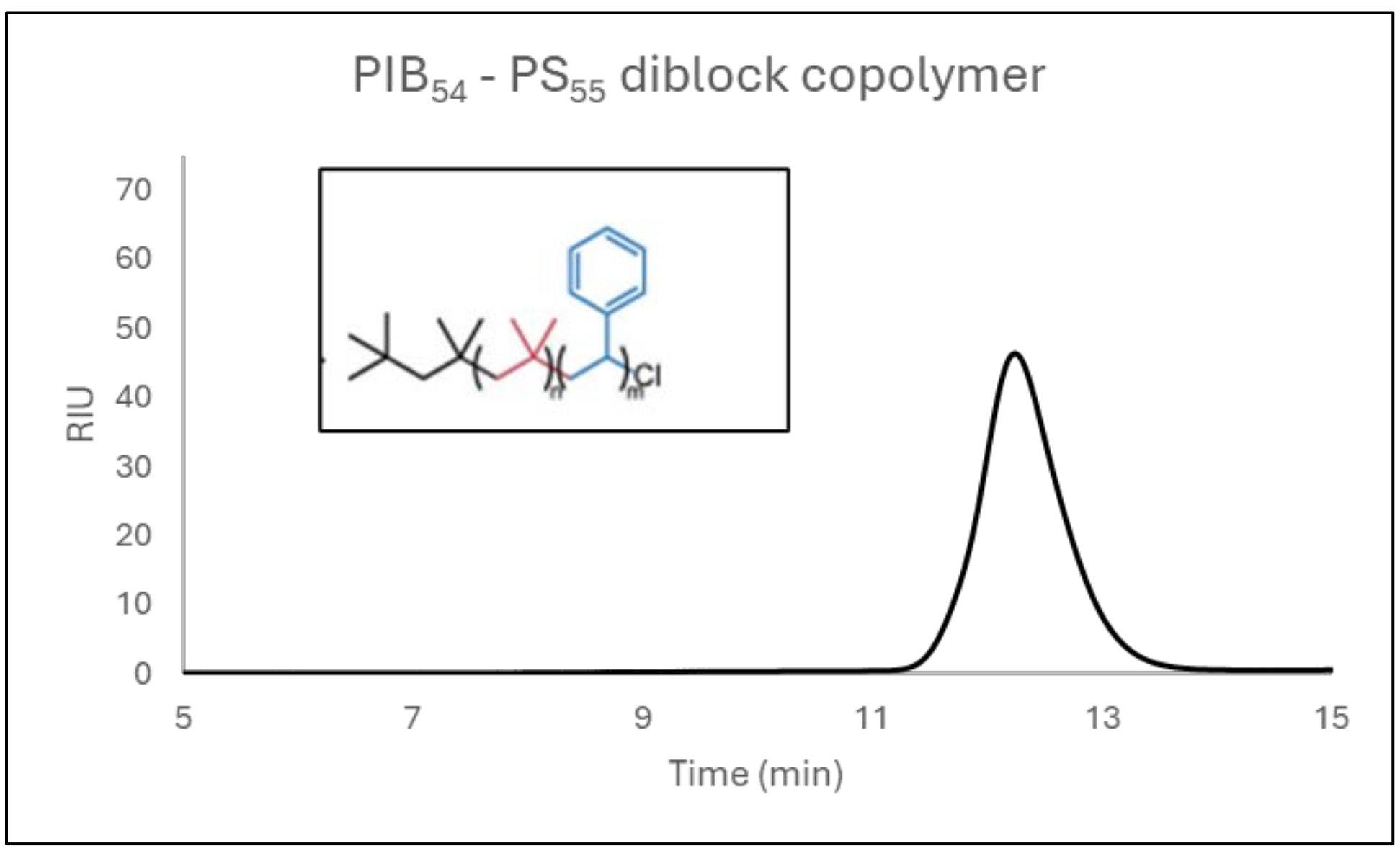

Figure S9: GPC trace for PIB – PS diblock copolymer showing monomodal distribution.

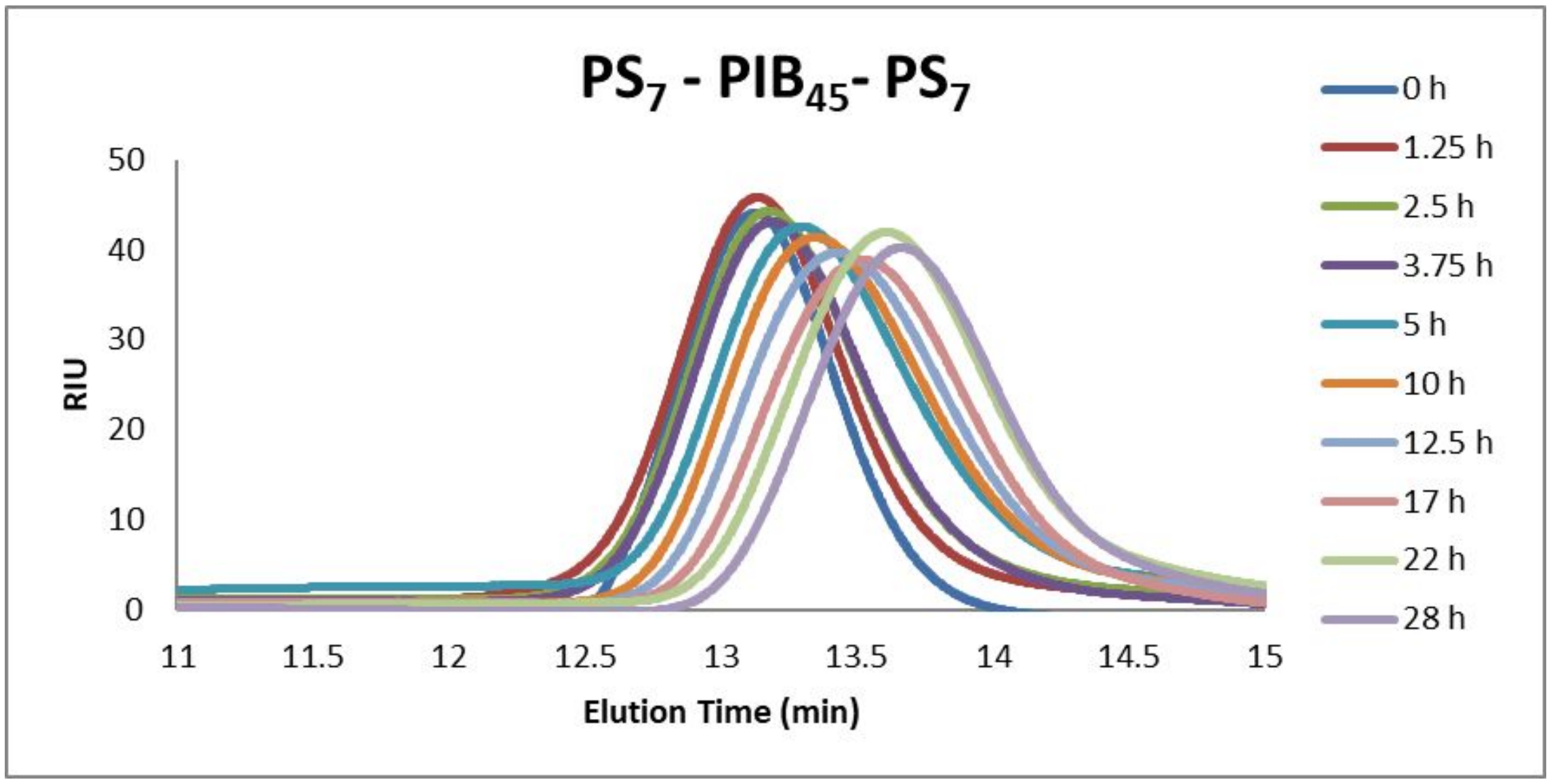

Figure S10: GPC overlay for PS_7PIB_{45}-PS_7 showing monomodal degradation pattern.

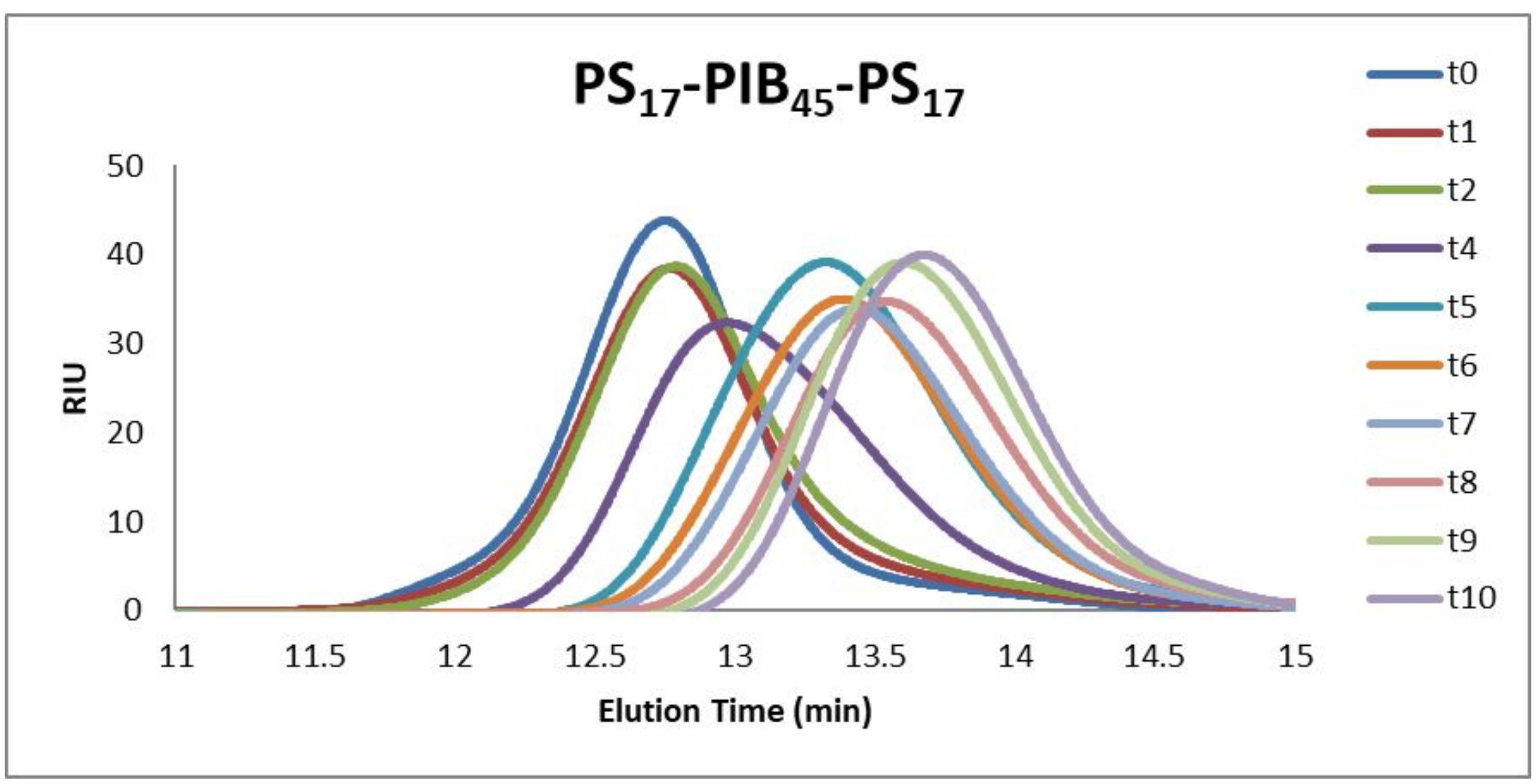

Figure S11: Molecular weight degradation in PS_{17}-PIB_{45}-PS_{17} showing monomodal chain scission.

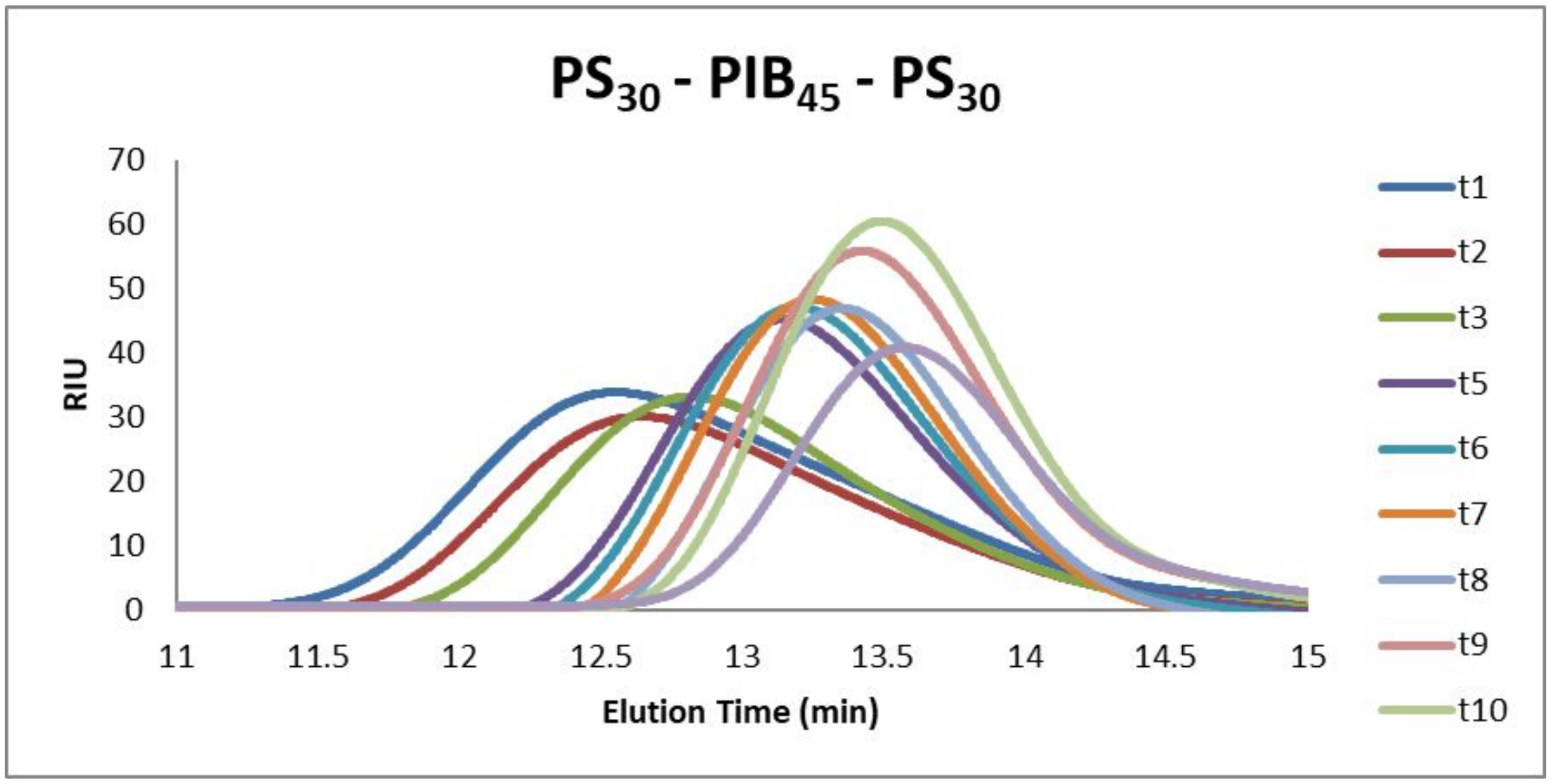

Figure S12: Molecular weight degradation in PS_{30}-PIB_{45}-PS_{30} showing monomodal chain scission.

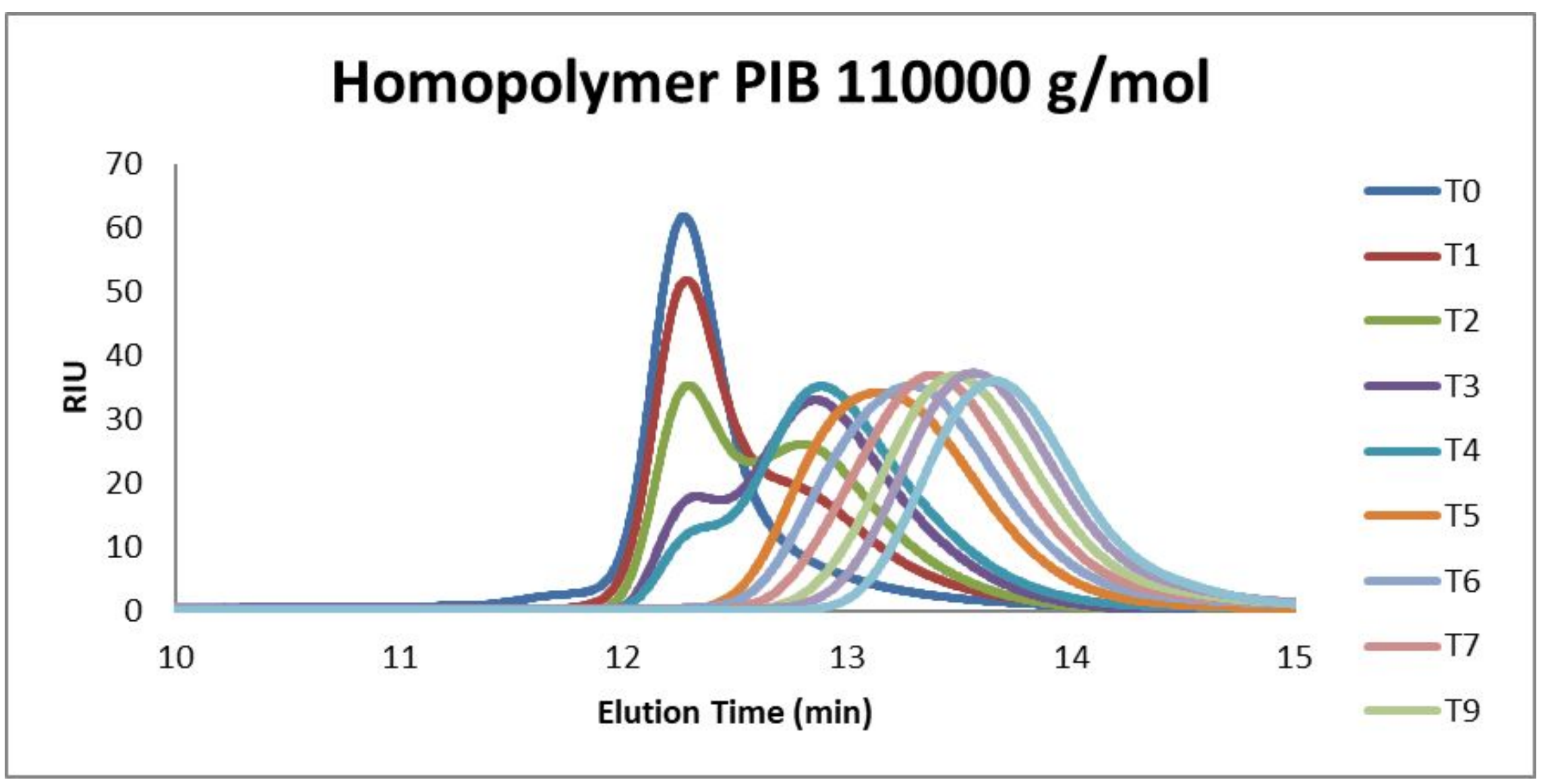

Figure S13: Molecular weight degradation during sonication of PIB homopolymer showing initial midpoint chain scission.

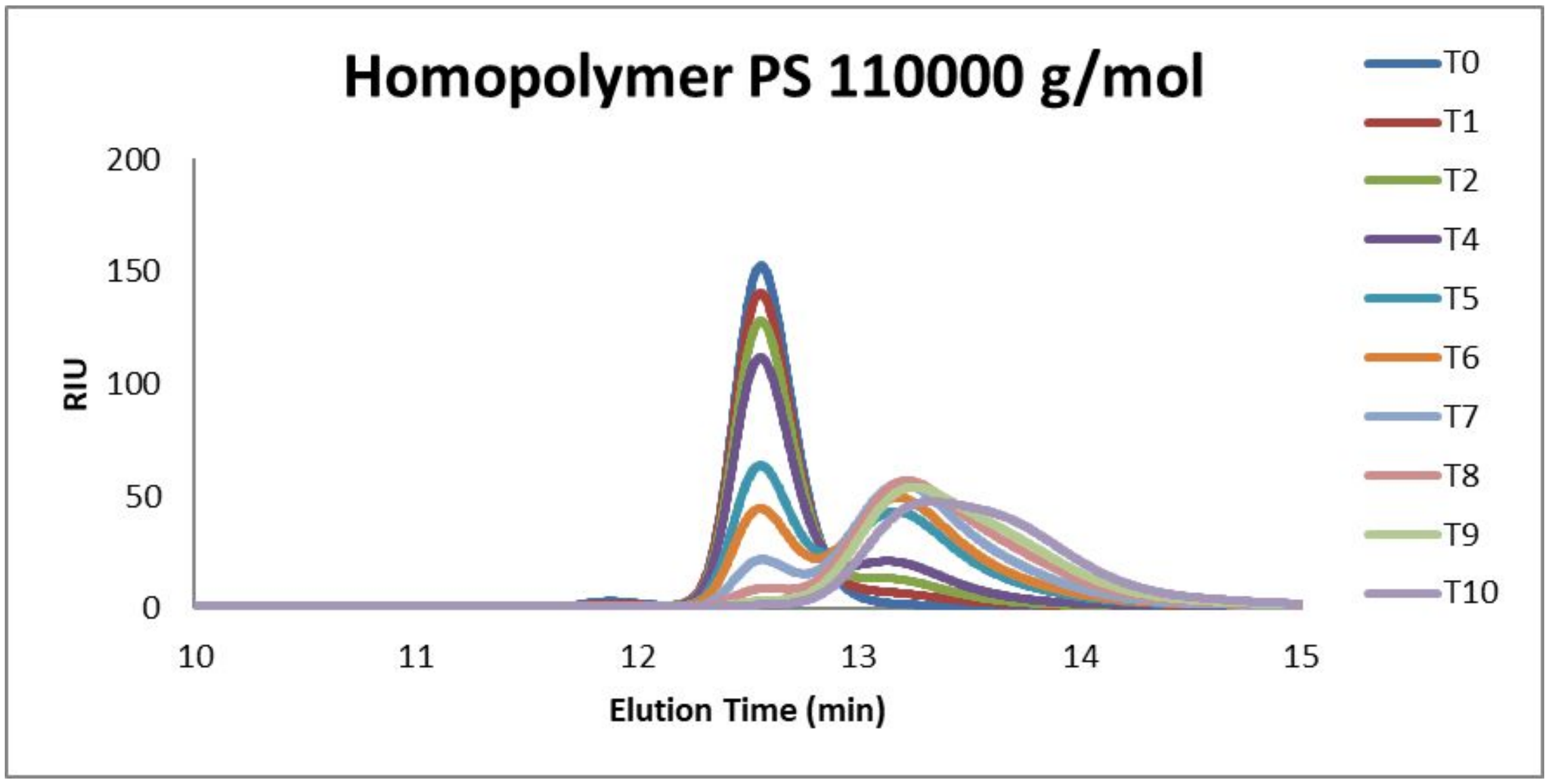

Figure S14: Molecular weight degradation during sonication of PS homopolymer showing initial midpoint chain scission.

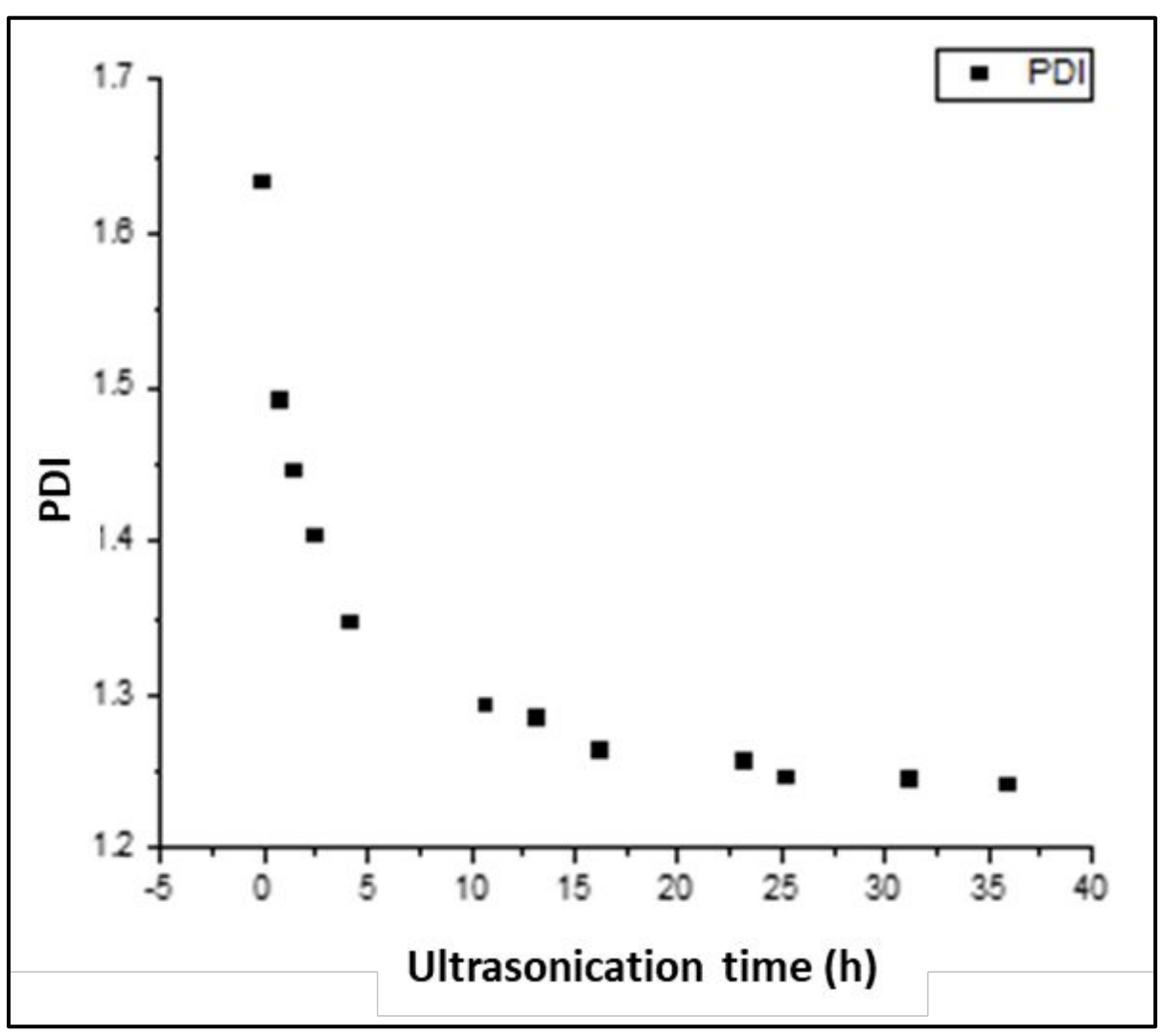

Figure S15: Polydispersity Index (PDI) as a function of time for PS_{30} –PIB_{45} -PS_{30} upon ultrasonication in THF at 2mg/ml: an exponential decay degradation profile.

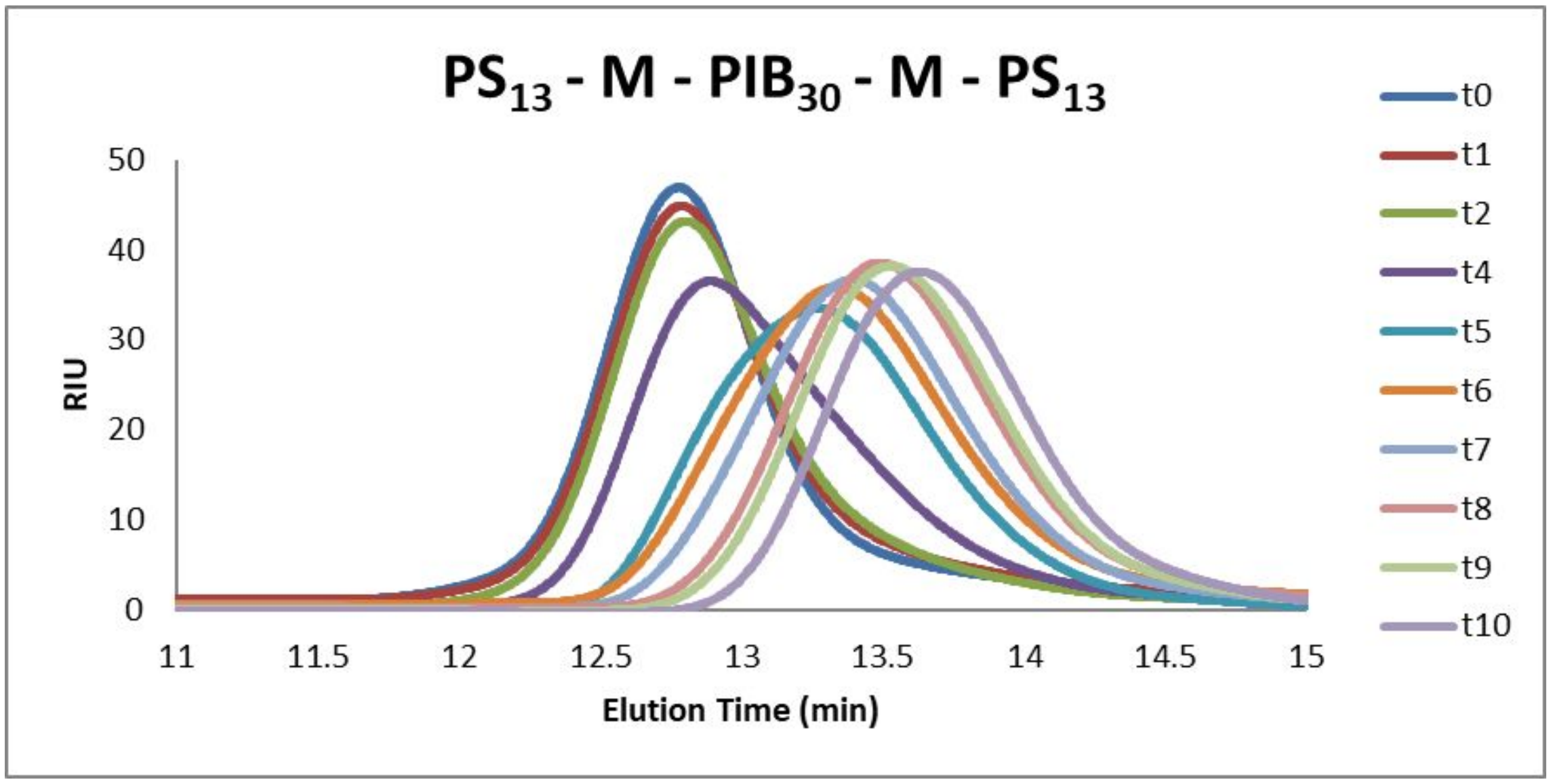

Figure S16: Molecular weight degradation in PS_{13}- M - PIB_{30}- M- PS_{13} showing monomodal chain scission.

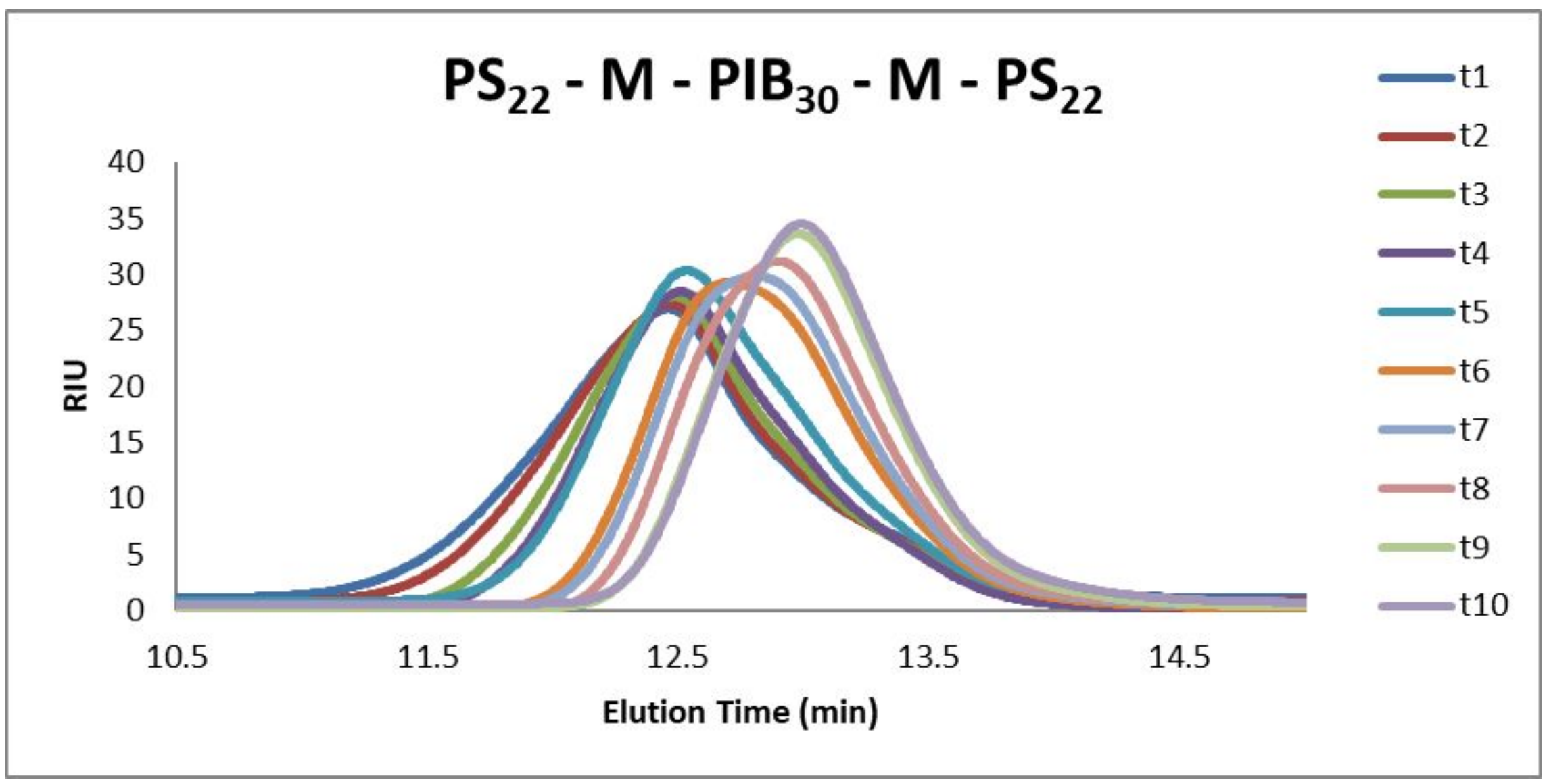

Figure S17: Molecular weight degradation in PS_{22}- M - PIB_{30}- M- PS_{22} showing monomodal chain scission.

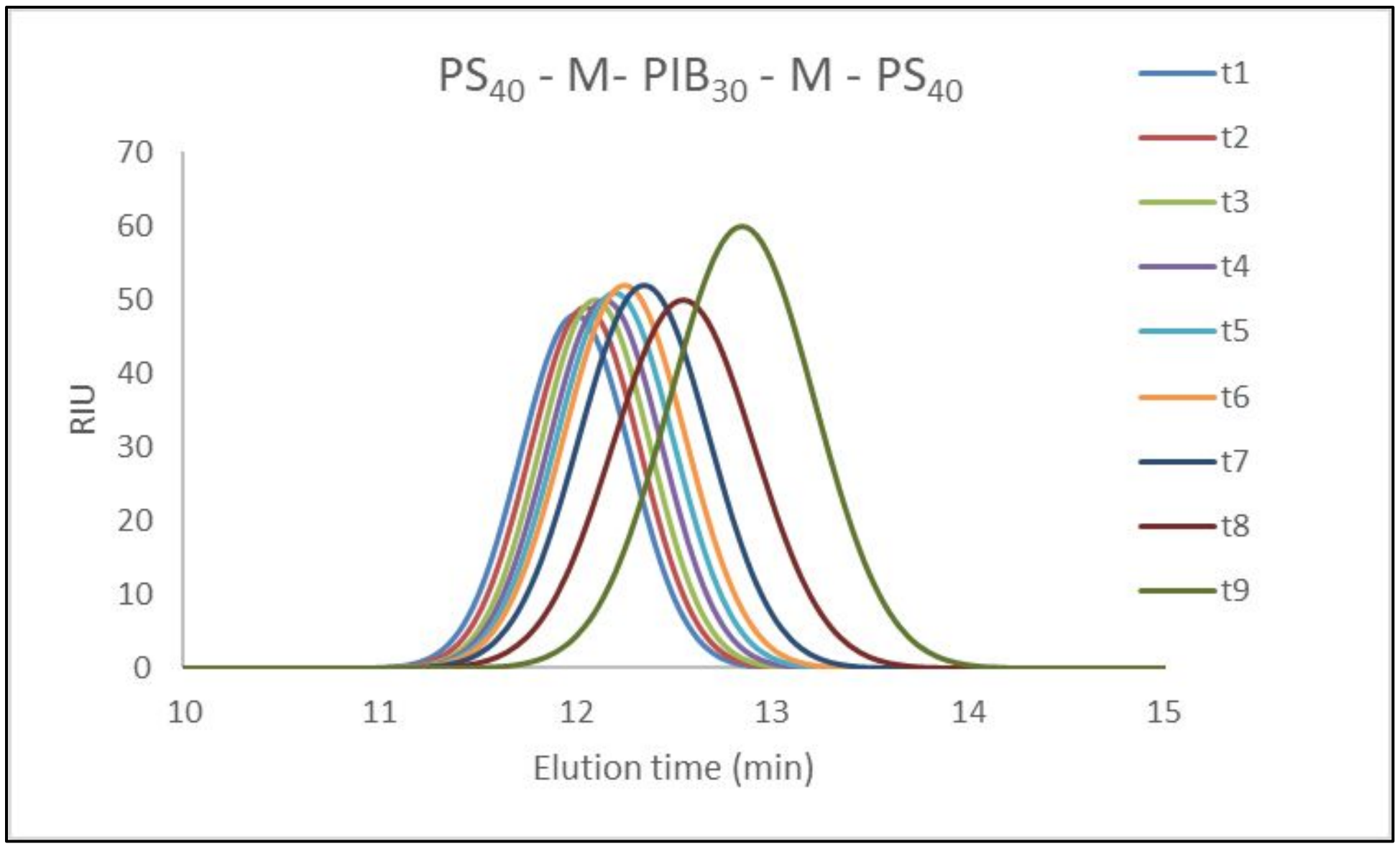

Figure S18: Molecular weight degradation in PS_{35}- M - PIB_{30}- M- PS_{35} showing monomodal chain scission.

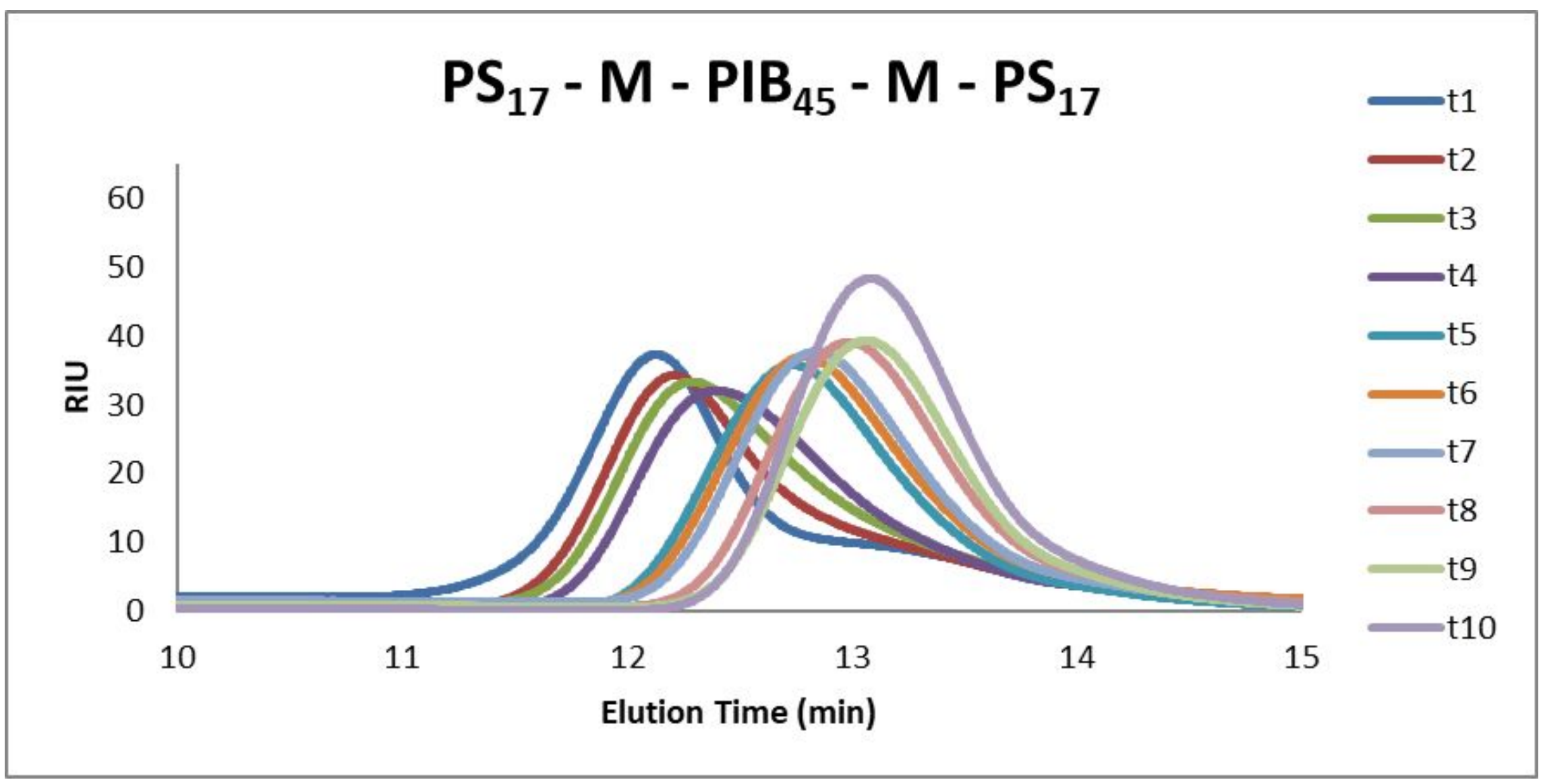

Figure S19: Molecular weight degradation in PS_{17}- M - PIB_{45}- M- PS_{17} showing monomodal chain scission.

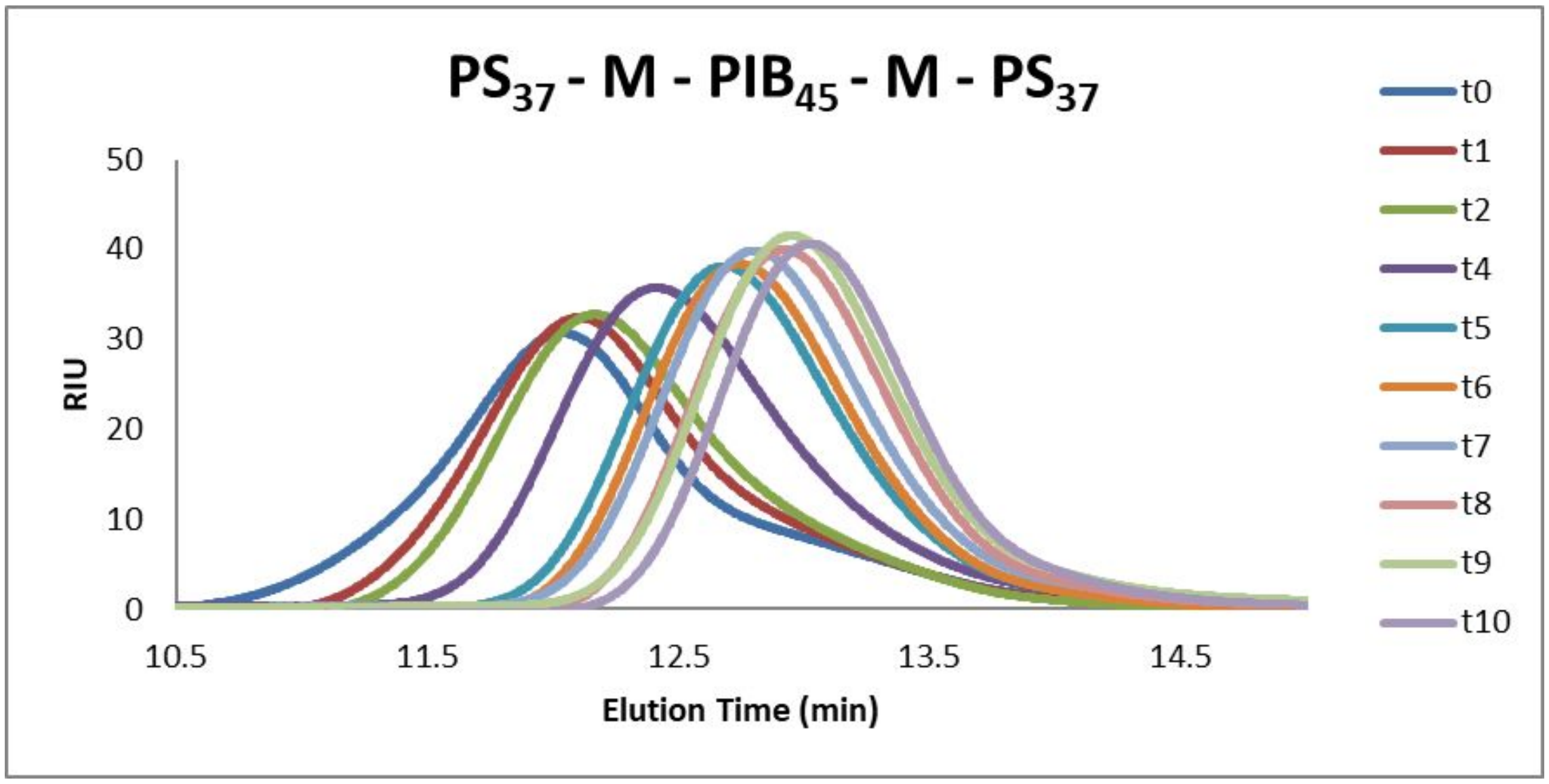

Figure S20: Molecular weight degradation in PS_{37}- M - PIB_{45}- M- PS_{37} showing monomodal chain scission.

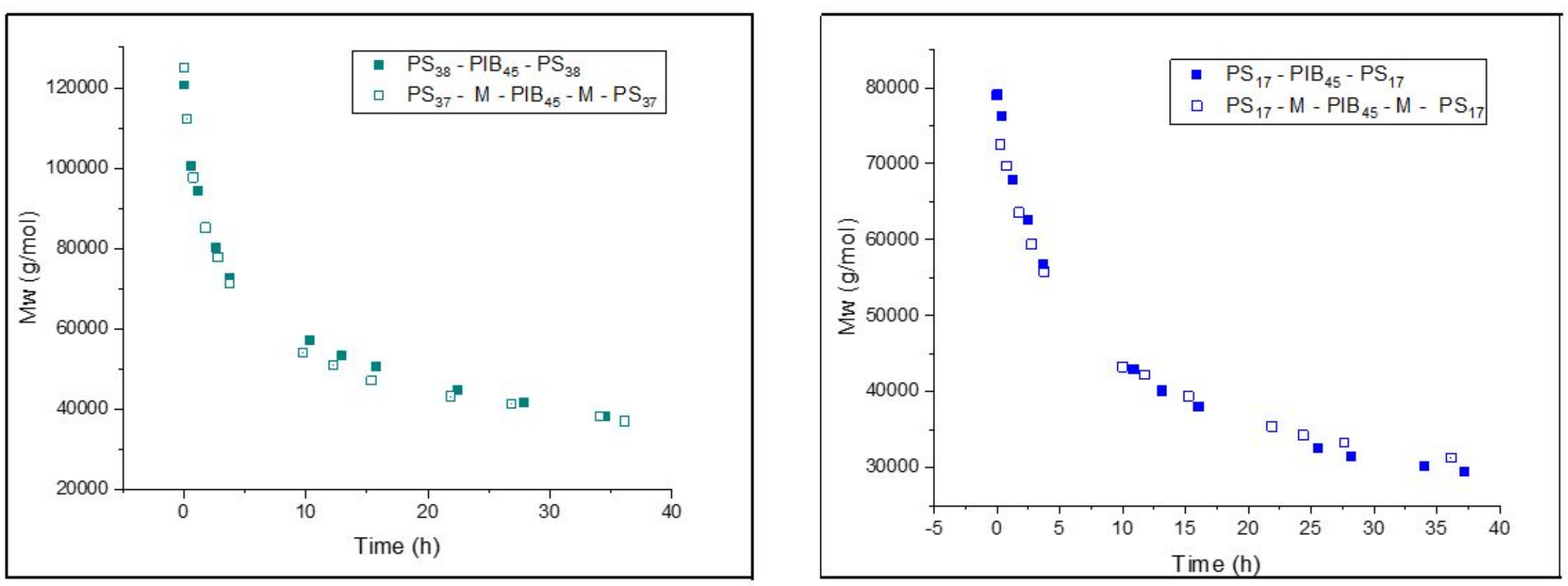

Figure S21: Comparison of molecular weight degradation of mechanophore and non-mechanophore containing triblocks with PIB midblock of 45Kg/mol.

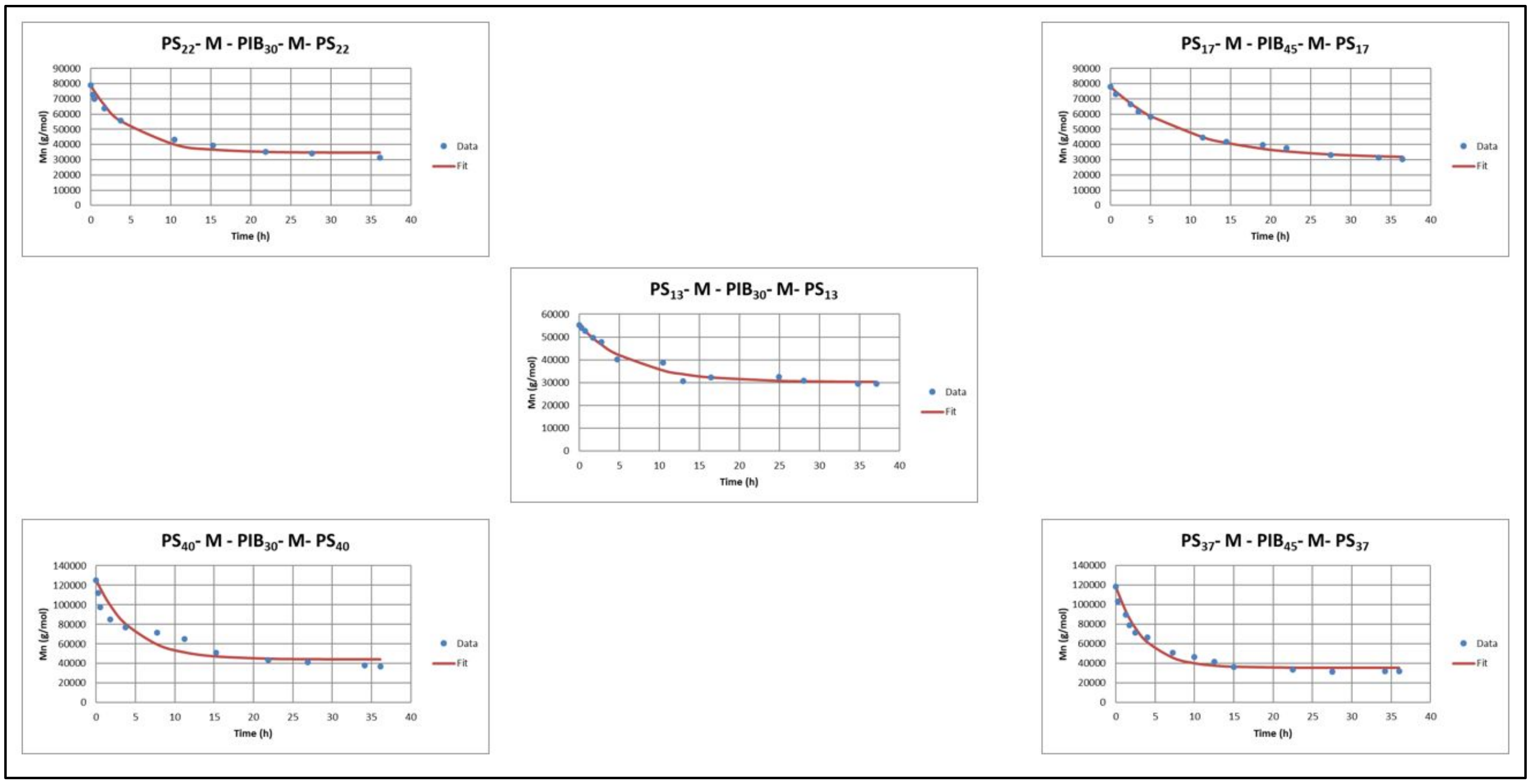

Figure S22: Baramboim model fits for mechanophore containing triblock samples

Table S2: Baramboim equation fit parameters for mechanophore containing triblocks

Sample	M_{w0}_(kgmol)	M_{lim}_(kgmol)	k_(1/h)	R2
PS_{22}- M - PIB_{30}- M- PS_{22}	79.10	34.71	0.2015	0.9737
PS_{45}- M - PIB_{30}- M- PS_{45}	115.1	44.04	0.2156	0.8873
PS_{13}- M - PIB_{30}- M- PS_{13}	55.32	30.16	0.1513	0.9751
PS_{37}- M - PIB_{45}- M- PS_{37}	118.3	35.34	0.2891	0.9709
PS_{17}- M - PIB_{45}- M- PS_{17}	78.00	31.03	0.1056	0.9933

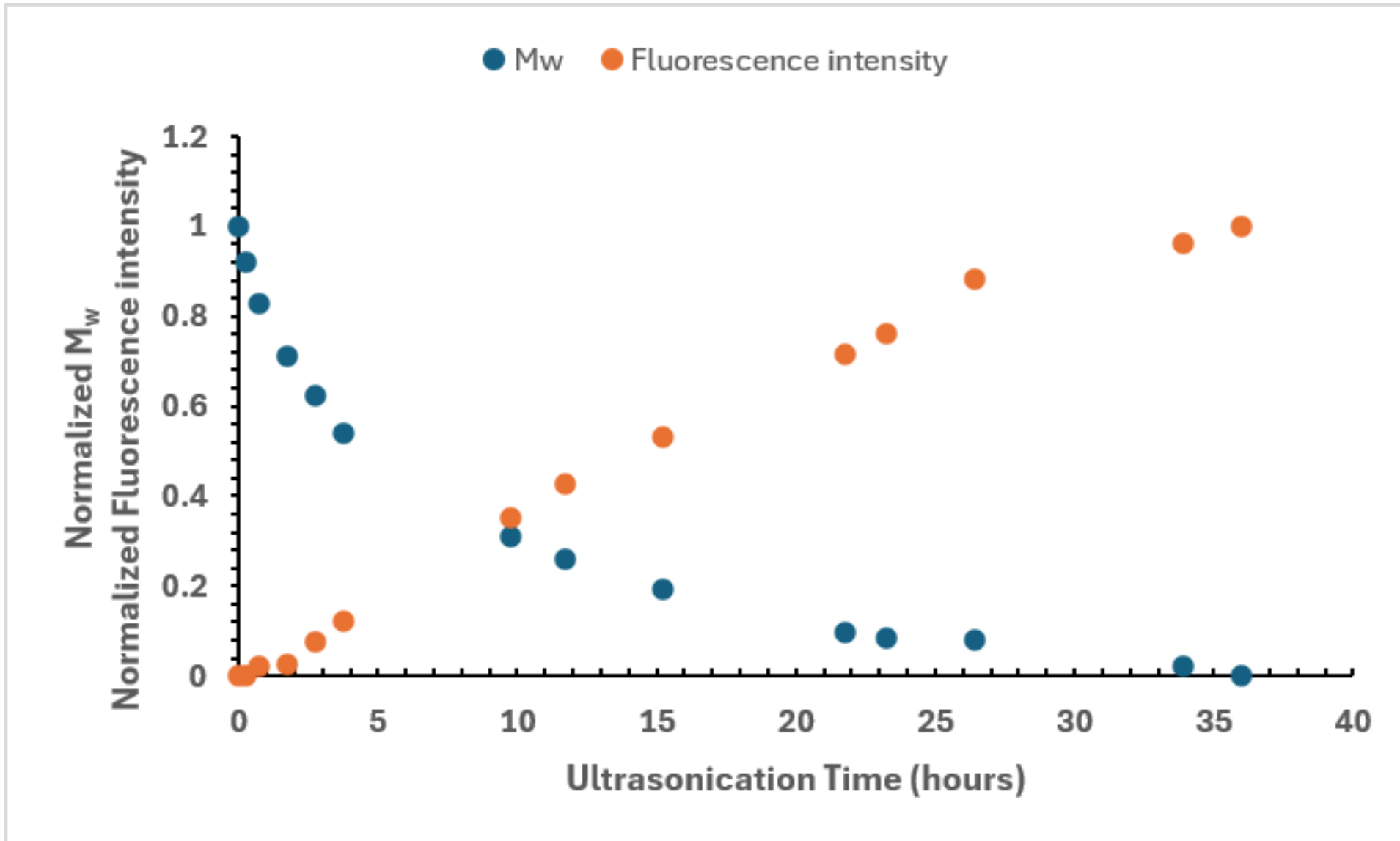

Figure S23: Normalized M_w and normalized fluorescence intensity as a function of ultrasonication time for PS_{22}-M-PIB_{30}-M-PS_{22} showing the difference in the mechanical chain scission and mechanophore activation behaviour in the initial stages and the latter stages of sonication.

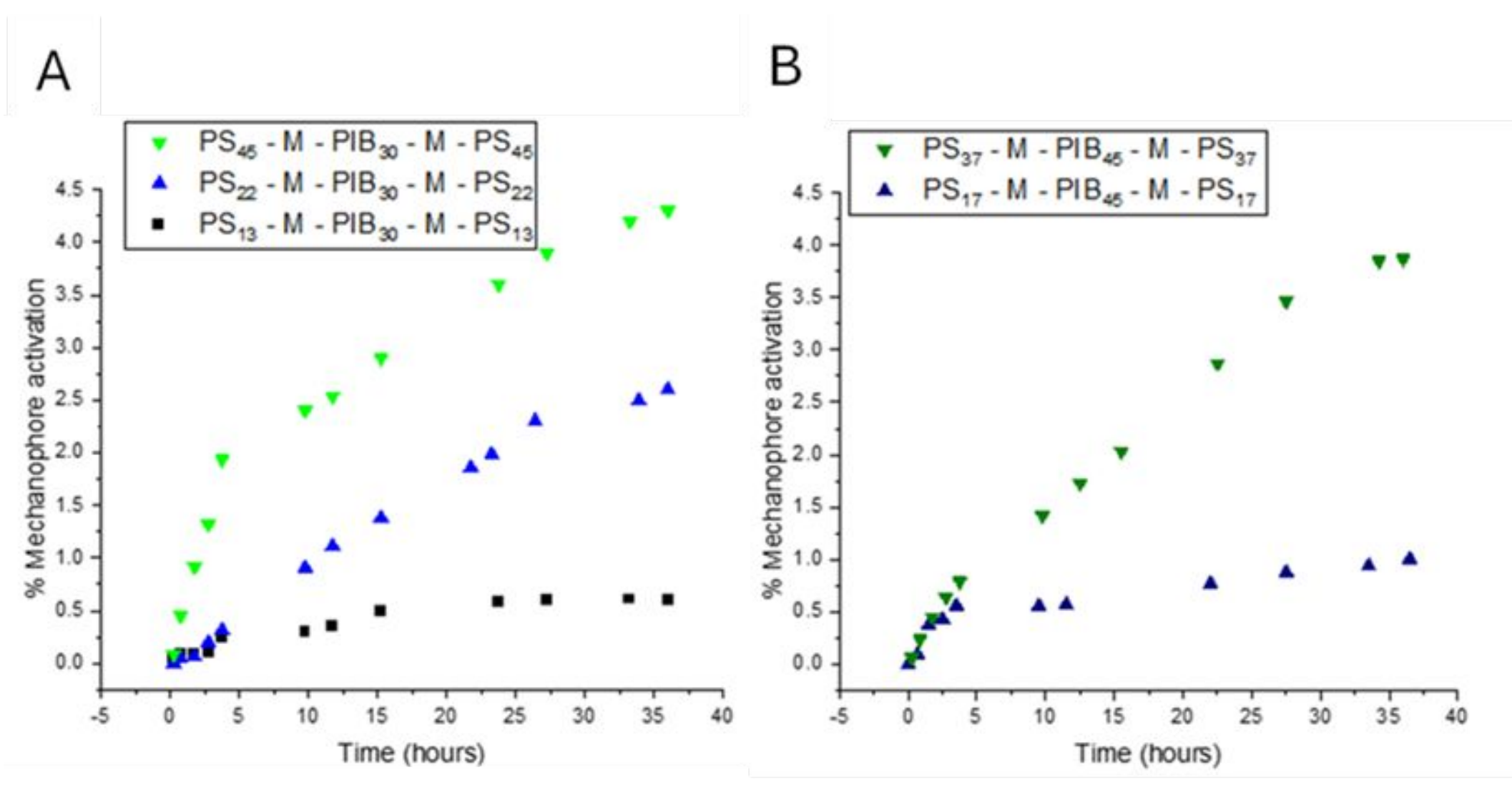

Figure S24: Mechanophore activation in the triblocks during ultrasonication A) 30 kg/mol PIB mid-block, B) 45 kg/mol PIB mid-block. Maximum standard deviation is ±0.1% within the 3 replicates tested for each triblock copolymer.

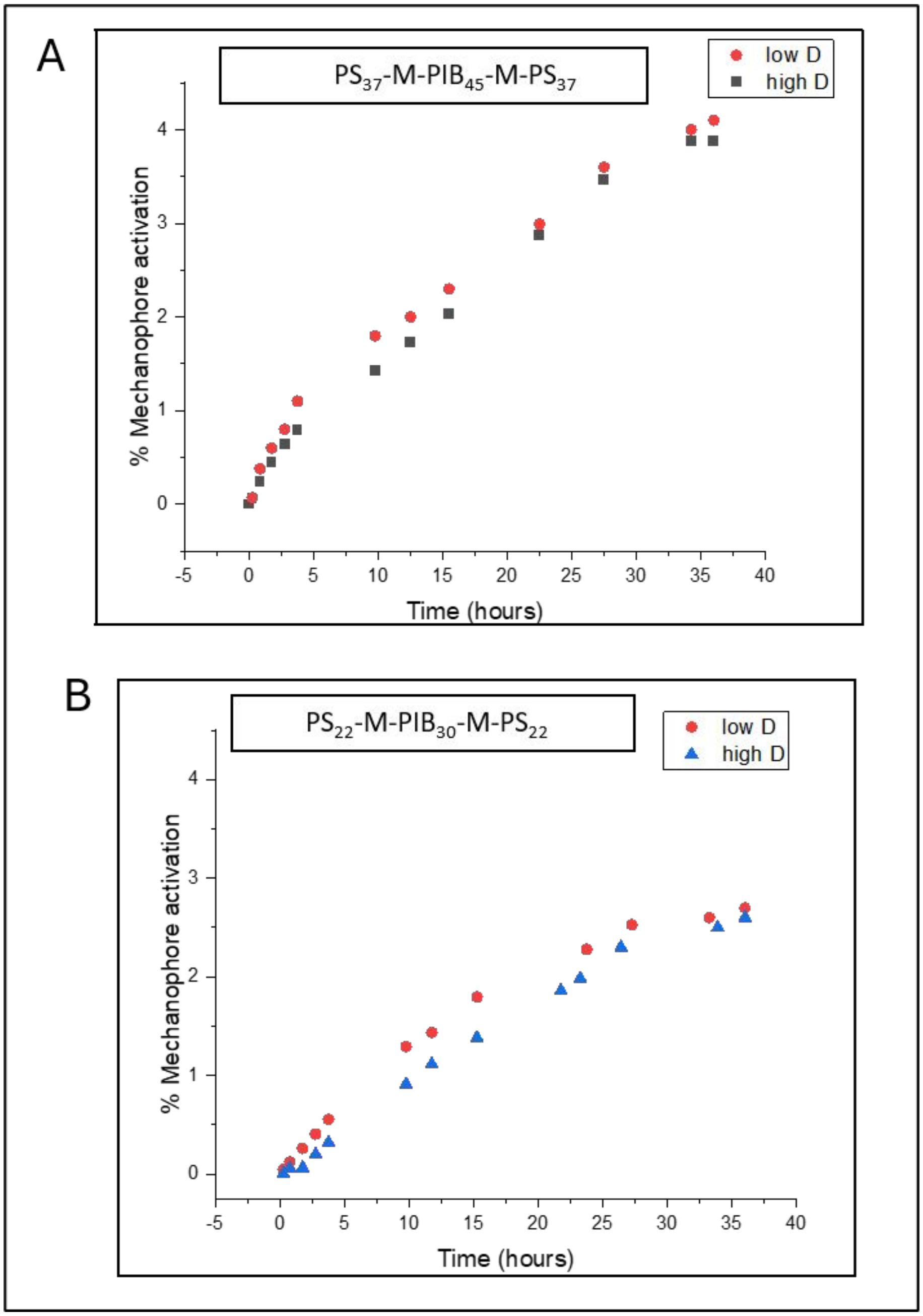

Figure S25: Comparison of average mechanophore activation as a function of sonication time for samples with low and high Đ for A) PS_{37}-M-PIB_{45}-M-PS_{37} and B) PS_{22}-M-PIB_{30}-M-PS_{22}

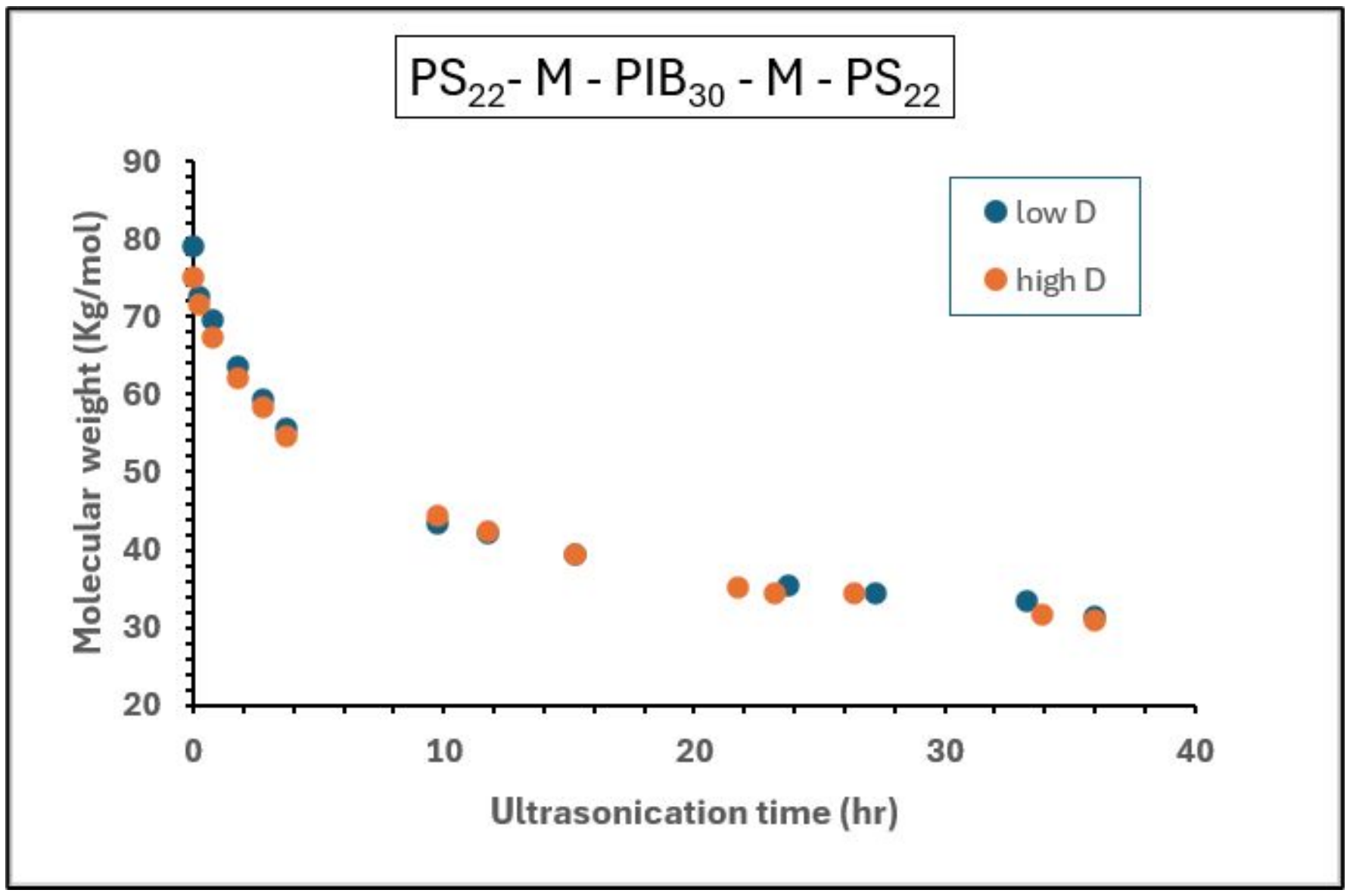

Figure S26: Molecular weight degradation curve during sonication for low and high *Đ* versions of PS_{22}-M-PIB_{30}-M-PS_{22}

Computational Details:

$$\Delta_r H^{\circ}(298K) = \sum H^{\circ}_{products} - \sum H^{\circ}_{reactants} \;\ldots\ldots\; Equation\ S1$$

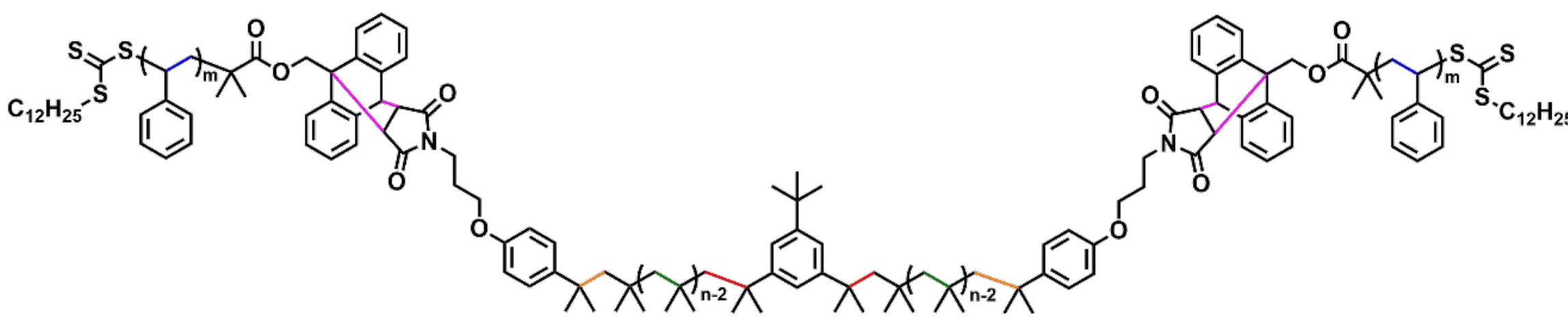

Figure S27. Chemical structure with colored bonds indicating regions of interest.

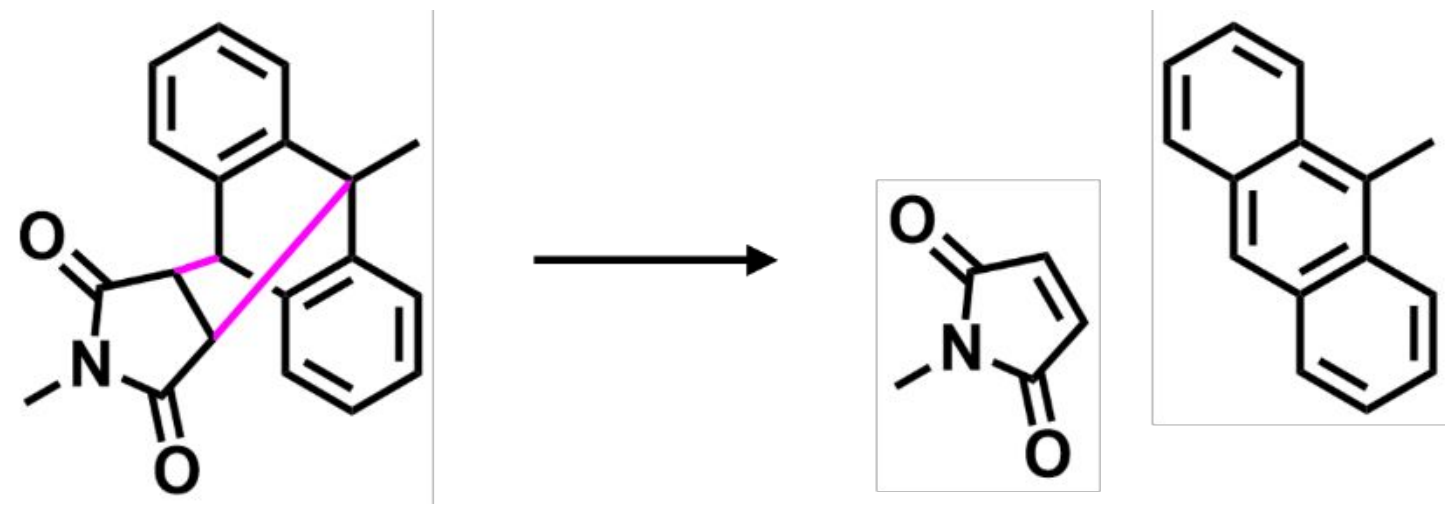

Figure S28: Structures used for the mechanophore moiety.

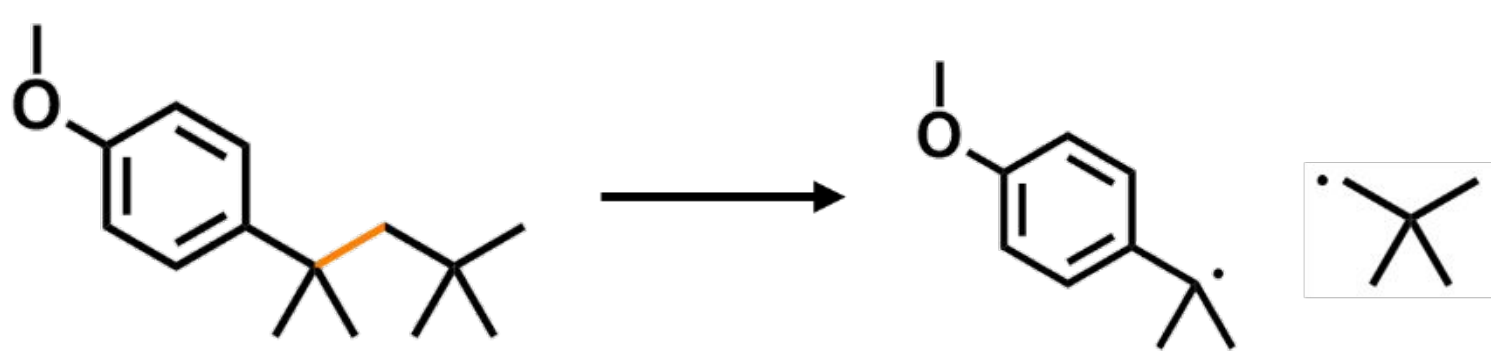

Figure S29: Structures used for the benzylic quaternary moiety at the point of addition of the alkoxybenzene quencher.

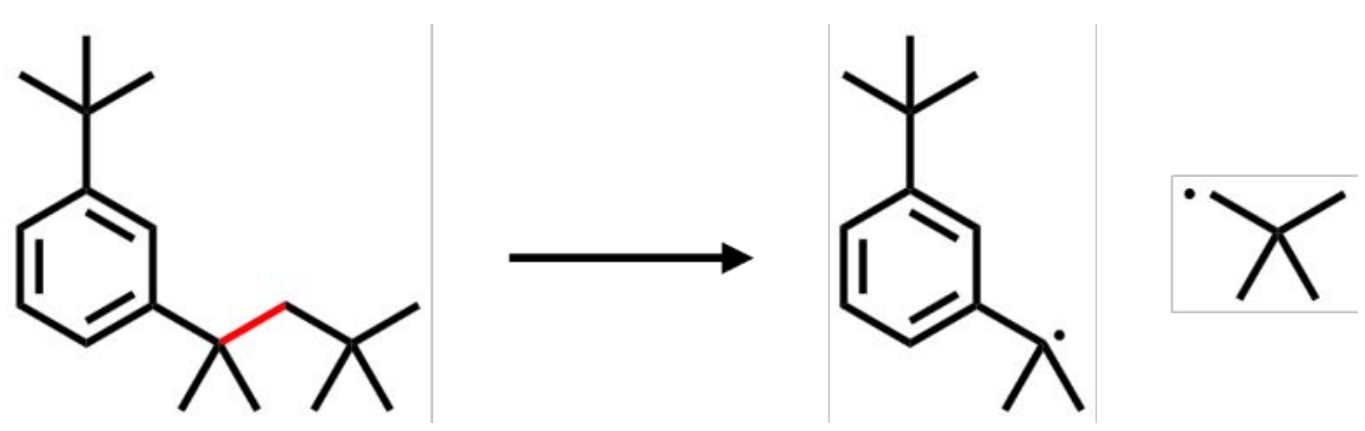

Figure S30: Structures used for the benzylic quaternary moiety at the bDCC initiator site.

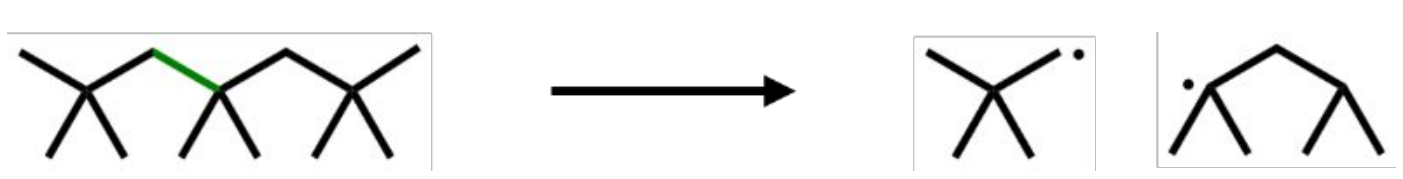

Figure S31: Structures used for polyisobutylene backbone moiety.

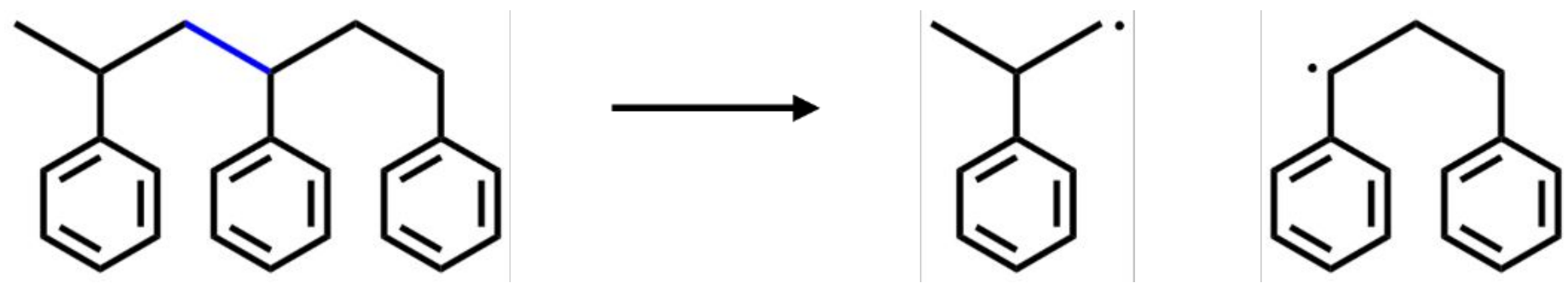

Figure S32: Structures used for polystyrene backbone moiety.

Table S3: Computationally calculated enthalpies of reaction for five regions of the copolymer that may break under ultrasonication

Chemical Moiety	$\Delta_r H^\circ$ (kJ/mol)
mechanophore	134
benzylic quaternary (quencher)	273
benzylic quaternary (initiator)	274
PIB backbone	298
PS backbone	310

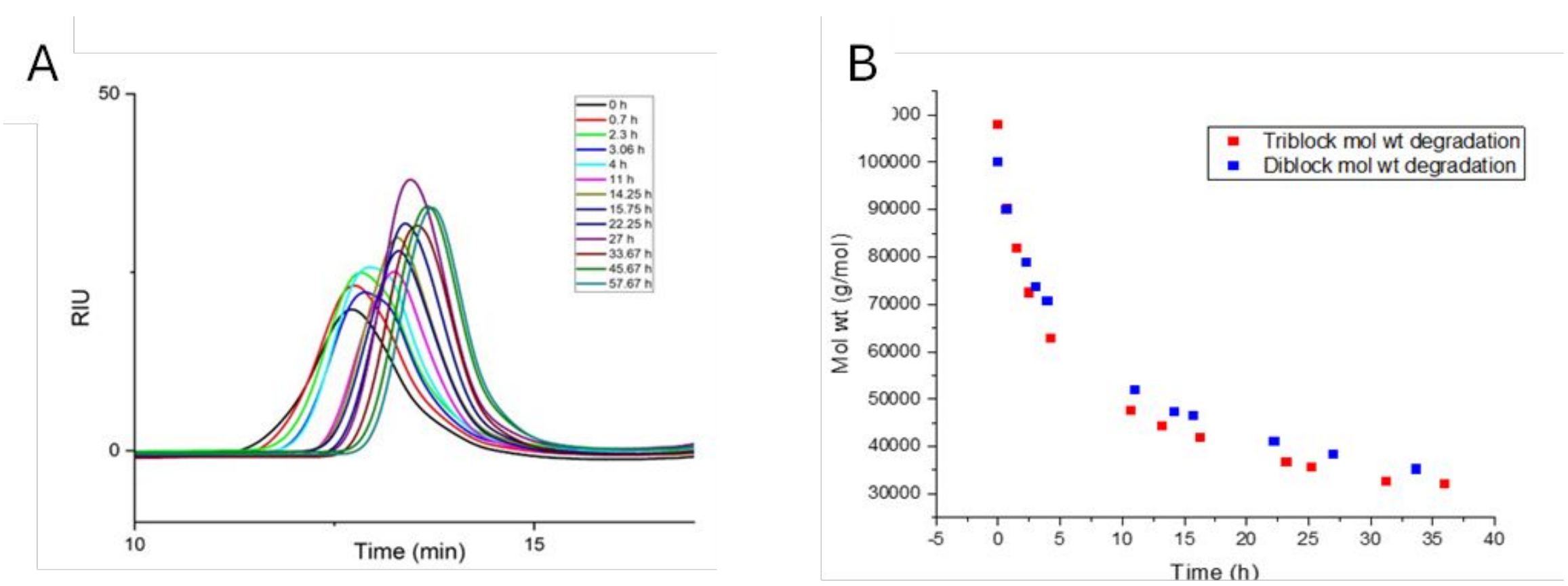

Figure S33: Molecular weight degradation for a PIB-PS diblock copolymer without the mechanophore and benzylic unit. A): Monomodal GPC curves for aliquots during ultrasonication. B) Comparison of molecular weight degradation of the diblock with a mechanophore-containing triblock copolymer of similar molecular weight.

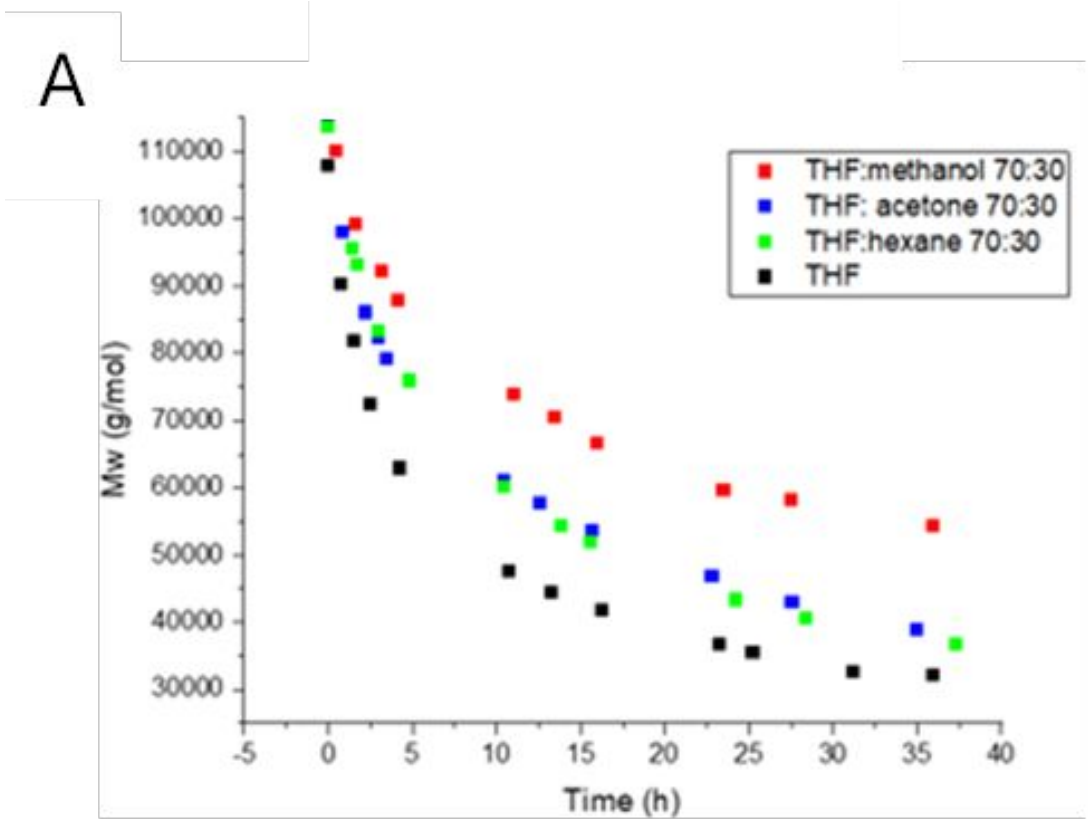

B

Solvent	M_{lim} (kg/mol)	Mechanophore activation (%)
THF	30	4.2%
THF:Acetone	42	1.9%
THF:Hexane	41	2%
THF:Methanol	53	1.4%

Figure S34: A) Molecular weight degradation of PS_{30}-PIB_{45}-PS_{30} in different solvent mixtures. B) Table showing mechanophore activation and limiting molecular weight after ultrasonication of PS_{37}-M-PIB_{45}-M-PS_{37} in different solvents (THF, acetone, hexanes, methanol)

Table S4: Solvent quality of 4 different solvents towards PIB and PS blocks.

	THF	Hexanes	Acetone	Methanol
PIB	Good solvent	Good solvent	Poor solvent	Poor solvent
PS	Good solvent	Poor solvent	Good solvent	Poor solvent

Table S.5: Baramboim equation fit parameters for PS_{30}-PIB_{45}-PS_{30} triblocks in various solvents

solvents	M_{w0} (kg/mol)	M_{lim} (kg/mol)	k (1/h)	R2
THF	107.9	32.11	0.2555	0.9765
THF:Methanol	115.1	56.66	0.1674	0.9934
THF:Hexane	113.9	37.74	0.1456	0.9512
THF:Acetone	113.7	40.29	0.1243	0.9858
